# CREBZF mRNA nanoparticles suppress breast cancer progression through a positive feedback loop boosted by circPAPD4

**DOI:** 10.1186/s13046-023-02701-5

**Published:** 2023-06-01

**Authors:** Boxuan Zhou, Jinhua Xue, Runxin Wu, Hongyu Meng, Ruixi Li, Zhaohong Mo, Hang Zhai, Xianyu Chen, Rongqiang Liu, Guie Lai, Xiaohong Chen, Taiyuan Li, Shiyang Zheng

**Affiliations:** 1 Department of General Surgery, The First Affiliated Hospital of Nanchang University, Nanchang, 330000 China; 2 Department of Breast Surgery, The First Affiliated Hospital of Gannan Medical University, Gannan Medical University, Ganzhou, 341000 China; 3 Department of Physiology, School of Basic Medical Sciences, Gannan Medical University, Ganzhou, 341000 China; 4 Zhongshan School of Medicine, Sun Yat-sen University, Guangzhou, 510080 China; 5 Department of Hepatobiliary Surgery, The Third Affiliated Hospital, Sun Yat-sen University, Guangzhou, 510630 China; 6 Department of Hepatobiliary and Pancreatic Surgery, The Eighth Affiliated Hospital, Sun Yat-sen University, Shenzhen, 518033 China; 7 Department of Hepatobiliary Surgery, Renmin Hospital of Wuhan University, Wuhan, 430060 China; 8 Department of Laboratory, The First Affiliated Hospital of Gannan Medical University, Ganzhou, 341000 China; 9 Department of Head and Neck surgery, Cancer Center of Guangzhou Medical University, Guangzhou, 510060 China

**Keywords:** Nanoparticles, Breast cancer, Targeted therapy, circRNAs, CREBZF, Feedback loop

## Abstract

**Background:**

Breast cancer (BC) negatively impacts the health of women worldwide. Circular RNAs (circRNAs) are a group of endogenous RNAs considered essential regulatory factor in BC tumorigenesis and progression. However, the underlying molecular mechanisms of circRNAs remain unclear.

**Methods:**

Expression levels of circPAPD4, miR-1269a, CREBZF, and ADAR1 in BC cell lines and tissues were measured using bioinformatics analysis, RT-qPCR, ISH, and IHC. Cell proliferation and apoptosis were measured using CCK8, EdU staining, flow cytometry, and TUNEL assays. Pearson correlation analysis, RNA pull-down, dual-luciferase reporter, and co-immunoprecipitation assays were used to explore the correlation among circPAPD4, miR-1269a, CREBZF, STAT3, and ADAR1. Effects of circPAPD4 overexpression on tumor progression were investigated using in vivo assays. Moreover, CREBZF mRNA delivered by polymeric nanoparticles (CREBZF-mRNA-NPs) was used to examine application value of our findings.

**Results:**

CircPAPD4 expression was low in BC tissues and cells. Functionally, circPAPD4 inhibited proliferation and promoted apoptosis in vitro and in vivo. Mechanistically, circPAPD4 biogenesis was regulated by ADAR1. And circPAPD4 promoted CREBZF expression by competitively binding to miR-1269a. More importantly, CREBZF promoted circPAPD4 expression by suppressing STAT3 dimerization and ADAR1 expression, revealing a novel positive feedback loop that curbed BC progression. Systematic delivery of CREBZF-mRNA-NPs effectively induced CREBZF expression and activated the positive feedback loop of circPAPD4/miR-1269a/CREBZF/STAT3/ADAR1, which might suppress BC progression in vitro and in vivo.

**Conclusion:**

Our findings firstly illustrated that circPAPD4/miR-1269a/CREBZF/STAT3/ADAR1 positive feedback loop mediated BC progression, and delivering CREBZF mRNA nanoparticles suppressed BC progression in vitro and in vivo, which might provide novel insights into therapeutic strategies for breast cancer.

**Supplementary Information:**

The online version contains supplementary material available at 10.1186/s13046-023-02701-5.

## Introduction

Breast cancer (BC) is the leading cause of cancer-associated deaths in women worldwide [1]. Despite substantial improvements in therapies including surgery, chemotherapy, radiotherapy, and targeted therapy, the mortality of BC remains high [2]. The predominant causes of poor BC prognosis are unpredictable progression, such as recurrence, metastasis, and drug resistance. Therefore, the molecular mechanisms underlying BC progression must be further investigated for novel effective therapies development.

Circular RNAs (circRNAs), newly discovered non-coding RNAs (ncRNAs), are generated by back-splicing and feature a covalently closed loop structure lacking 5′- and 3′-ends [3, 4]. Compared with the precursor linear mRNA, circRNAs are commonly expressed at lower levels, and are consecutively resistant to exonuclease degradation [5, 6]. Mounting evidence has revealed that aberrant circRNAs expression patterns play critical oncogenic as well as anti-cancer roles in multiple cancers [7, 8]. For instance, CircSEC62 accelerates microvascular invasion via activating NOTCH1/Snail pathway in hepatocellular carcinoma (HCC) [9]. While circDLC1 is expressed at low levels in HCC tissues and represses HCC malignancy by combining with HuR [10]. Importantly, circRNAs usually bind miRNAs to modulate the levels of downstream proteins, acting as competing endogenous RNA (ceRNA) and participating in tumor progression [11].

The regulation of circRNA biogenesis is the focus of current research [3, 4, 12]. Adenosine deaminase acting on RNA 1 (ADAR1) is closely related with circRNA formation through its adenosine-to-inosine (A-to-I) RNA editing, which decreased circRNAs production by lowering the complementarity of circRNA precursor’s flanking introns [13, 14]. Notably, the biological role and formation of circRNAs were also modulated by IL6-STAT3 pathway [15, 16]. For instance, the biogenesis of circRNA GGNBP2 (cGGNBP2) was mediated by IL-6/STAT3 pathway activation, and IL-6/cGGNBP2-184aa (a protein encoded by cGGNBP2)/STAT3 formed a positive feedback loop to promote tumor progression for intrahepatic cholangiocarcinoma. Furthermore, IL6-STAT3-ADAR1 interplay promotes oncogenicity in multiple myeloma [17]. Therefore, it is important to elucidate whether there is a mechanism in STAT3-ADAR1 pathway regulating circRNAs biogenesis in BC.

CREBZF, a member of ATF b-ZIP/CREB family of transcription factors, usually binds b-ZIP response elements to fulfill its function. Recent studies reported that the dysregulation of CREBZF regulated the development of several cancers including melanoma, medulloblastoma, gastric adenocarcinoma, and osteosarcoma, by affecting tumor cell proliferation and apoptosis [18–20]. More importantly, CREBZF inhibits dimerization and activation of STAT3 pathway, leading to reduction of cell cycle progression [21]. Nevertheless, whether CREBZF mediates STAT3 pathway activation to affect the biogenesis of circRNAs in BC cells remains mysterious.

Nanoparticles (NPs), 1-1000 nm diameter ultra-small particles, have been considered a promising material for nucleic acids delivery in clinical applications [22, 23]. The encapsulation or combination of nucleic acid by NPs via electrostatic interactions or chemical conjugation can overcome the nucleic acids degradation in circulation [24]. Moreover, NP carriers can be engineered to respond to acidic environments, such as in solid tumors or within endosomes inside cells, to release their cargo on-demand [25]. Importantly, owing to low toxicity and high tumor enrichment of NPs, various studies believe that NPs for nucleic acid delivery might be promising type of therapeutic for cancer treatment [24, 26].

In this study, we identified a novel circRNA, circPAPD4, which consisted of the exon 2, 3, and 4 of the PAPD4 gene. We firstly found that circPAPD4 was low-expressed in BC, and regulated by ADAR1. CircPAPD4 curbed the proliferation and promoted apoptosis of BC by sponging miR-1269a to regulate CREBZF expression. Besides, we demonstrated that CREBZF suppressed ADAR1 expression via blocking STAT3 dimerization. These results revealed a positive feedback loop consisting of circPAPD4, miR-1269a, CREBZF, STAT3, and ADAR1 that plays an important part on BC progression. Furthermore, we introduced NPs to deliver CREBZF-mRNA and successfully suppressed the proliferation and accelerated apoptosis of BC by activating of CREBZF/STAT3/ADAR1/circPAPD4/miR-1269a positive feedback loop. These findings would help provide new perspectives on the molecular mechanism of BC progression and insights for the development of novel therapies.

## Materials and methods

The detailed procedures of RNA extraction and RT-qPCR, RNase R assay, actinomycin D assay, subcellular fractionation, fluorescence in situ hybridization (FISH), western blotting, co-immunoprecipitation, CCK-8 assay, EdU staining, analyses of apoptosis by flow cytometry, TUNEL assay, RNA pull-down assay, RIP assay, luciferase reporter assay, immunohistochemical (IHC) staining, RNA in situ hybridization (ISH), immunofluorescence, in vitro STAT3 dimerization assay, preparation of CREBZF mRNA NPs, physicochemical features and constancy of mRNA NPs in serum, pharmacokinetic (PK) study in vivo, biodistribution of CREBZF-mRNA-NPs in BC model, and bioinformation analysis are described in Supplementary Experimental Procedures.

### Tissue collection and ethical approval

143 BC patients were enrolled for clinical specimen collection from 2007 to 2012 at the First Affiliated Hospital of Gannan Medical University. None of the patients was received any adjuvant therapy before surgery. Additionally, 20 paracancerous breast tissues from those patients were enrolled as normal group. Necessarily, the research was carried out following the guidelines of the Declaration of Helsinki, and all patients provided written consent after being informed about the study’s details, as well as the aforementioned declaration and informed consents. This study had obtained the ethics approvals from the Committee for Ethical Review of the First Affiliated Hospital of Gannan Medical University.

### Cell culture

Human breast cancer cell lines MCF-7, SKBR-3, BT474, BT549, MDA-MB-468, and normal breast epithelial cell line MCF-10 A were acquired from American Type Culture Collection (ATCC). Cancer cells were incubated in Dulbecco’s Modified Eagle’s Medium (DMEM) (Gibco, USA) with 10% fetal bovine serum (FBS) (Gibco, USA). MCF-10 A cells were incubated in DMEM/F12 (Invitrogen, USA), 5% horse serum (Gibco, USA), EGF (20 ng/ml), hydrocortisone (0.5 mg/ml), cholera Toxin (100 ng/ml), and insulin (10 µg/ml). All these cells were cultured at 37℃ circumstance with 5% CO_2_.

### Lentiviral vector construction and transfection

The lentivirus transduction and efficacy assessment were conducted on the basis of previous study [27]. The lentivirus-based vector for circPAPD4 overexpression and corresponding negative control vectors were constructed by GeneRay (Shanghai, China). After lentivirus transduced into breast cancer cells, stably transduced cells were selected by puromycin (2 µg/mL). Additionally, overexpression plasmid for circPAPD4, Flag-CREBZF, Myc-STAT3 and His-STAT3, short hairpin RNA plasmids of ADAR1 and CREBZF (sh-ADAR-1, sh-ADAR-2, sh-CREBZF-1, sh-CREBZF-2), overexpression plasmid of CREBZF and STAT3 (OE-CREBZF, OE-STAT3), miR-1269a mimics, and miR-1269a inhibitors were also constructed by GeneRay (Shanghai, China). Transfection was performed by using Lipofectamine® 3000 (Invitrogen; Thermo Fisher Scientific, USA) followed by the manufacturer’s instructions. All the sequences of this section were listed in supplementary Table S1-3.

### Xenograft experiments

Six-week-old female BALB/C nude mice were housed under specific pathogen-free conditions in the Center of Laboratory Animals of Sun Yat-sen University. BC cells transduced with circPAPD4 overexpression or control vector were orthotopically implanted into the mammary fat pad of mice. To evaluate the antitumor efficacy of CREBZF-mRNA-NPs in vivo, nude mice with CREBZF-null xenograft tumor were constructed as demonstrated above. When the tumors grow to 150 mm^3^ (day8), mice were randomly revived PBS, control NPs, or CREBZF-mRNA-NPs through the tail vein every 3 days for 7 cycles. At day 32, mice were sacrificed. The xenograft and major organs were collected for immunofluorescence and hematoxylin-eosin (H&E) staining. Blood biochemical indicators, including alanine transaminase (ALT), aspartate transaminase (AST), creatinine (Cr), and blood urea nitrogen (BUN), were also measured using corresponding kits. Tumor volume was calculated using follows formula: volume (mm^3^) = (shortest diameter)^2^ × (longest diameter)/2. Data of tumor volume and body weight were recorded every 4 days. Importantly, xenograft experiments were confirmed by the Institutional Animal Care and Use Committee of Sun Yat-sen University and were done according to institutional ethical guidelines on animal care.

### Statistical analysis

SPSS 23.0 (SPSS, Chicago, IL, USA) and GraphPad (Prism ver. 7, GraphPad Prism Software, La Jolla, CA, USA) were used to carry out statistical analysis. All the data are expressed as mean ± SD from three independent experiments. Paired, two-tailed Student’s t tests were used to analysis the non-parametric data between breast cancer samples and their paracancerous tissues. Unpaired, two-tailed Student’s t tests or one-way ANOVA were used to compare other parametric data. The correlation between circPAPD4 expression and clinicopathological characteristics was evaluated by employing Chi-square or Fisher exact tests. Survival curves were established on the basis of the Kaplan-Meier method, and log-rank test was used to describe statistical differences. The Cox proportional hazards model applied on univariate and multivariate analyses was utilized to pinpoint significant prognostic factors for recurrence-free survival (RFS). Pearson correlation analysis was used to assess genes expression correlations. Statistically significant differences were defined as *p* < 0.05.

## Results

### circPAPD4 expression is low in breast cancer

**Fig. 1:**
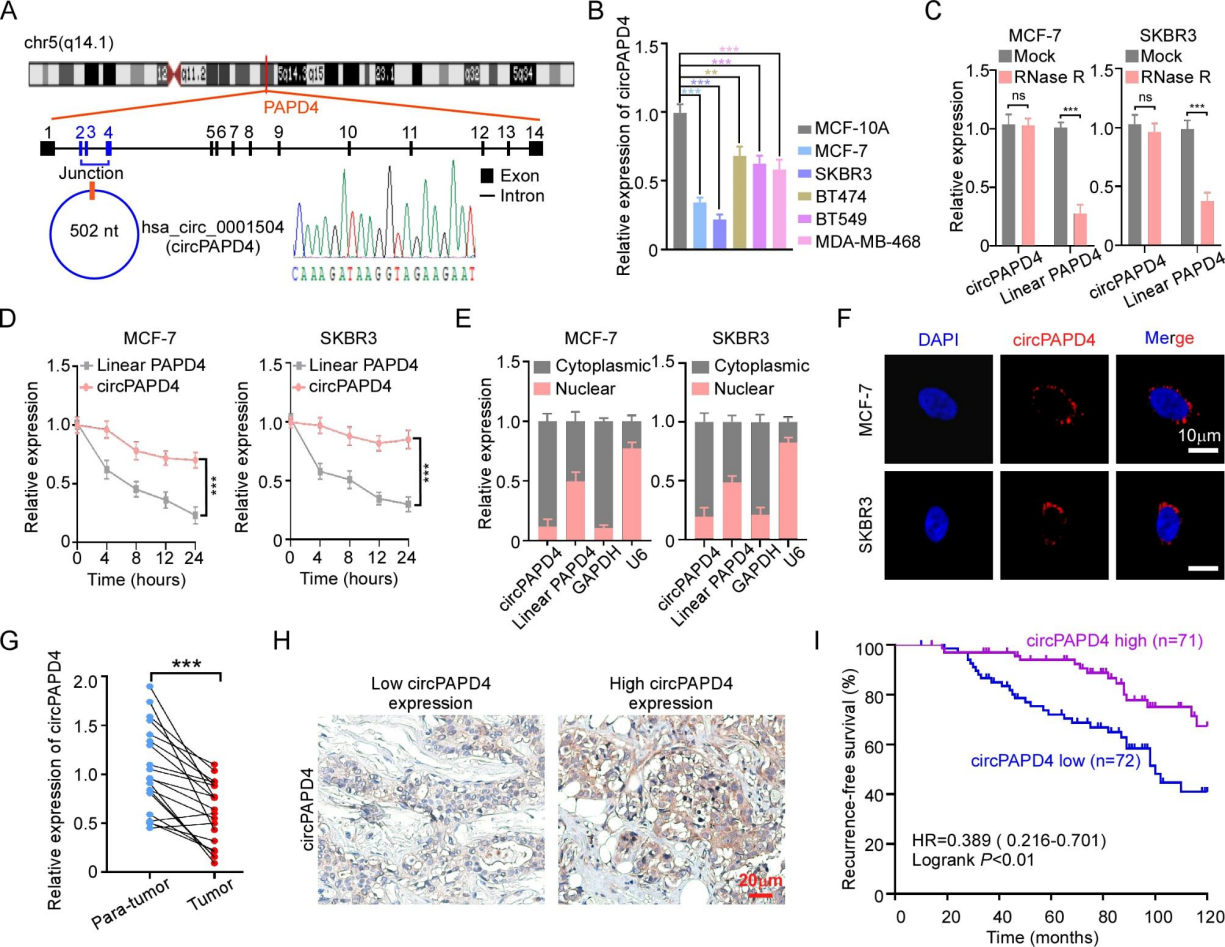
circPAPD4 is down-regulated in BC and correlated with favorable prognosis. **(A)** Diagram of circPAPD4 formation. The back-splicing junction of circPAPD4 was verified by Sanger sequencing. **(B)** Relative expression of circPAPD4 in MCF-10A and breast cancer cell lines (MCF-7, SKBR3, BT474, BT549, and MDA-MB-468) were examined by RT-qPCR. **(C)** Relative expression of circPAPD4 and linear PAPD4 in MCF-7 and SKBR3 cells with or without RNase R treatment were examined by RT-qPCR. **(D)** After treated with Actinomycin D, the half-life period of circPAPD4 and linear PAPD4 in MCF-7 and SKBR3 cells were analyzed by RT-qPCR. **(E)** The main distribution of circPAPD4 and linear PAPD4 in MCF-7 and SKBR3 cells was measured by RT-qPCR after subcellular fractionation. **(F)** FISH assay was used to determine the subcellular distribution of circPAPD4 in MCF-7 and SKBR3 cells. Scale bar: 10 μm. **(G)** circPAPD4 expression levels in 20 pairs BC tissues and paracancerous tissues were measured by RT-qPCR. **(H)** Representative images of ISH of the high/low expression circPAPD4 in BC tissues. Scale bar: 20 μm. **(I)** The association between circPAPD4 expression and recurrence rate of BC patients was illustrated using Kaplan-Meier log-rank method. Data are presented as means ± SD. **p<0.01;***p < 0.001.

To investigate the expression pattern of circRNAs in BC, two microarray datasets (GSE182471 and GSE165884) were downloaded from the Gene Expression Omnibus (GEO). By intersecting the top 50 differentially down-regulated circRNAs in each microarray (Table S4-S5), we identified six circRNAs that were significantly down-regulated in BC (Fig. S1). Among them, hsa_circ_0001504 (circPAPD4) was the most conserved and chosen for the further study (Table S6). Based on the UCSC Genome Browser Home (http://genome.ucsc.edu/), circPAPD4 (chr5:78915434–78919312) was arose from exon 2, 3, and 4 of PAPD4 gene by back-splicing; the head-to-tail splicing of circPAPD4 was verified by Sanger sequencing (Fig. 1A). A panel of BC cell lines and the mammary gland epithelial cell line MCF-10 A were used to investigate the expression of circPAPD4 by RT-qPCR. Significant downregulation of circPAPD4 in BC cell lines was found compared to that in MCF-10 A cells (Fig. 1B). RNase R and Actinomycin D treatment confirmed that the circular structure of circPAPD4 made it more stable than linear PAPD4 (Fig. 1C-D). Subcellular fractionation and FISH assays revealed that circPAPD4 was mainly distributed in the BC cells cytoplasm (Fig. 1E-F).

RT-qPCR results on 20 pairs of BC tissues and paracancerous tissues indicated that circPAPD4 expression was down-regulated in BC tissues (Fig. 1G). To evaluate the correlation between circPAPD4 expression and clinicopathological characteristics in 143 BC patients, we divided those patients into high/low circPAPD4 group based on the median expression level of circPAPD4 measured by ISH (Fig. 1H). Low circPAPD4 expression was substantially correlated with larger tumor size, advanced tumor-node-metastasis (TNM) stages, and higher Ki-67 expression of BC tumors (Table S7). In addition, BC patients with lower circPAPD4 expression presented an unfavorable RFS relative to those with higher circPAPD4 expression as determined using Kaplan–Meier analysis (Fig. 1I). In Cox multivariable regression analysis, circPAPD4 expression served as an independent prognostic factor for RFS in BC (Table S8). Collectively, these results suggest that low circPAPD4 expression may contribute to BC progression.

### CircPAPD4 overexpression inhibits proliferation and promotes apoptosis of breast cancer cells in vitro and in vivo

**Fig. 2:**
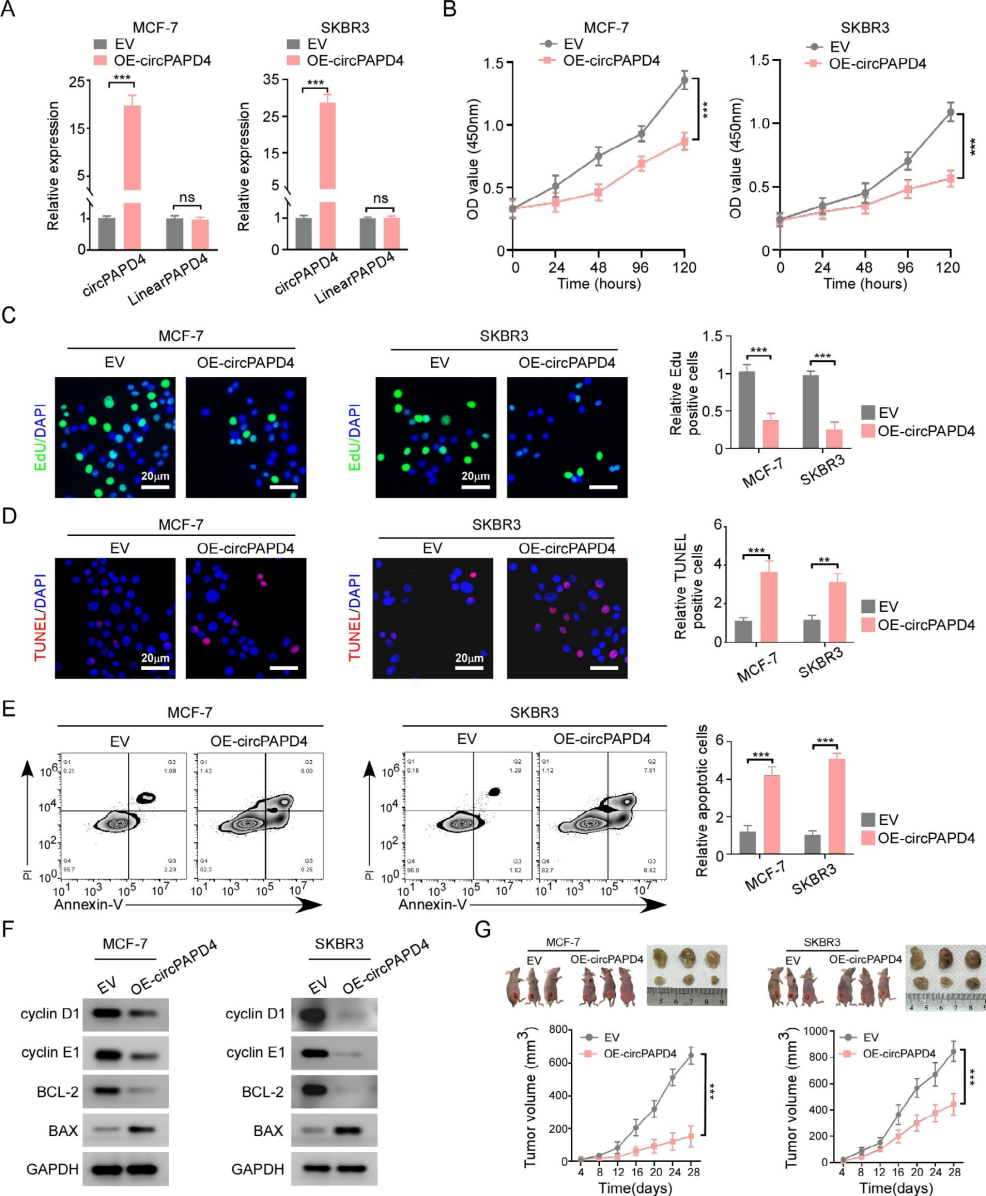
Overexpression of circPAPD4 inhibits proliferation and promotes apoptosis for BC cells. **(A)** After transduced with circPAPD4 overexpression vector (OE-circPAPD4) or empty vector (EV), circPAPD4 and linear PAPD4 mRNA expression in MCF-7 and SKBR3 cells were analyzed by RT-qPCR. **(B-C)** CCK-8 and EdU assays were used to measure proliferation ability of BC cells in indicated groups; Scale bar: 20 μm. **(D-E)** TUNEL and flow cytometry analyses were applied to evaluate the apoptosis of BC cells in indicated groups; Scale bar: 20 μm. **(F**) The expression of cyclin-D1, cyclin-E1, BCL-2, and BAX in indicated groups were evaluated by western blotting. **(G)** Tumor volume of BALB/c nude mice was inoculated with BC cells stably transduced with OE-circPAPD4 or EV. n = 3 mice each group. Data are presented as means ± SD. **p<0.01; ***p < 0.001.

To investigate the biological functions of circPAPD4 in BC cells, MCF-7 and SKBR3 cells were transduced with circPAPD4 overexpression lentiviral vector (OE-circPAPD4) or empty vector (EV). RT-qPCR assay was used to validate the transduction efficiency. Meanwhile, overexpression of circPAPD4 did not alter PAPD4 mRNA expression (Fig. 2A). CCK8 and EdU assays revealed that upregulation of circPAPD4 significantly inhibited BC cells proliferation (Fig. 2B-C). TUNEL and flow cytometry assays demonstrated that circPAPD4 overexpression induced BC cells apoptosis (Fig. 2D-E). To further clarify circPAPD4 overexpression impacts BC cells proliferation and apoptosis, we investigated the expression of cell cycle checkpoint proteins (cyclin D1 and cyclin E1), and apoptosis-related proteins (BCL-2 and BAX) using western blotting. We found that circPAPD4 overexpression substantially inhibited the expression of cyclin D1, cyclin E1, and BCL-2, while enhancing BAX expression (Fig. 2F). Additionally, circPAPD4 overexpression notably decreased the growth of xenograft tumors in nude mice compared with that in the EV group (Fig. 2G). Collectively, these data indicate that circPAPD4 exerts anti-tumor effects in breast cancer in vitro and *in mice*.

### ADAR1 regulates the expression of circPAPD4

**Fig. 3:**
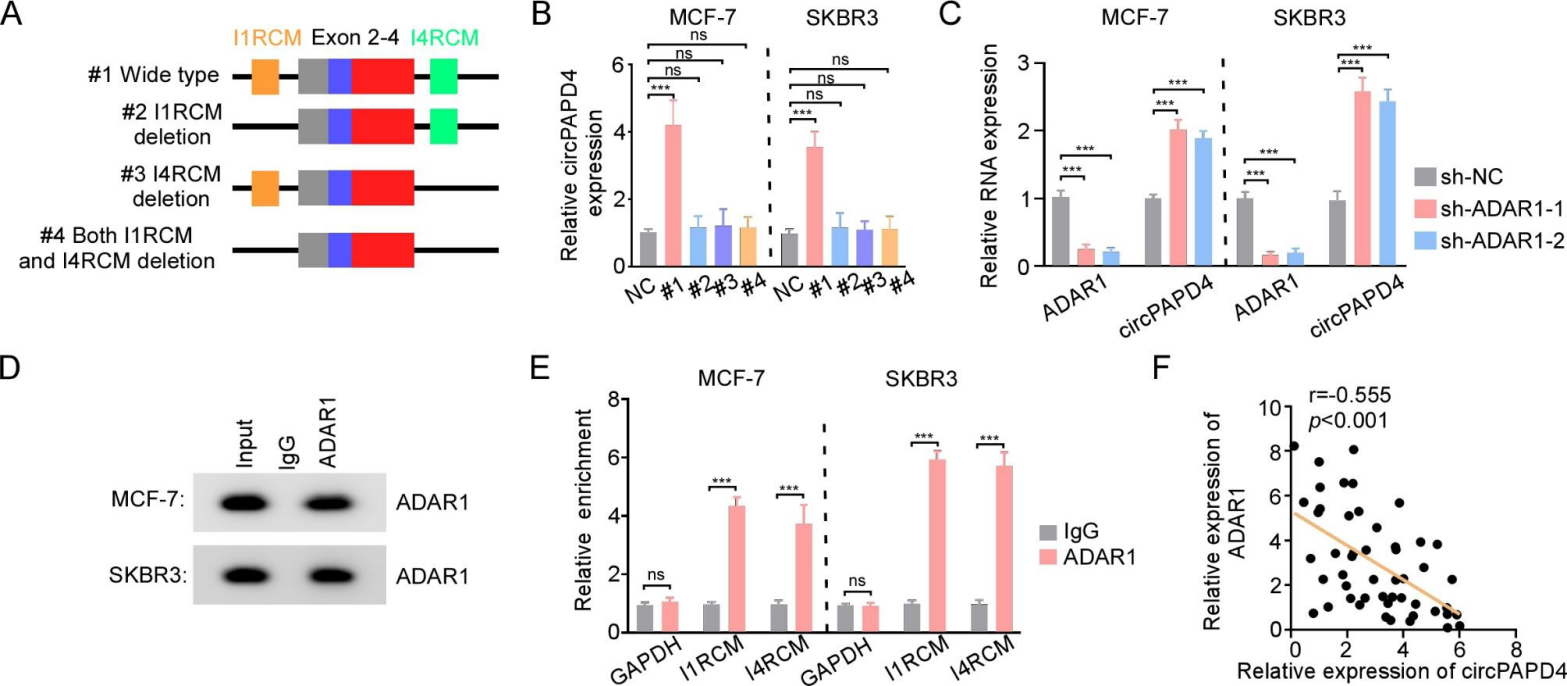
ADAR1 regulates the biogenesis of circPAPD4 via binding to the flanking inverted complementary sequences. **(A)** Diagram indicated the construction of four types of vectors (#1, #2, #3, and #4) with or without the flanking inverted complementary sequences. **(B)** Relative circPAPD4 expression was assessed by RT-qPCR in MCF-7 and SKBR3 cells transfected with four types of vectors or NC. **(C)** RT-qPCR was used to assess relative ADAR1 and circPAPD4 expression in BC cells transfected with the sh-ADAR1-1, sh-ADAR1-2, or sh-NC. **(D-E)** Anti-ADAR1 antibody was used to performed RNA immunoprecipitation for assessment of endogenous ADAR1 binding to RNA in BC cells. RT-qPCR was used to evaluate the combining relationship between ADAR1 and I1RCM/I4RCM. **(F)** The correlation between ADAR1 mRNA and circPAPD4 expression in 50 BC tissues was measured using Pearson’s Correlation analysis. Data were presented as means ± SD. ***p < 0.001.

Since reverse complementary matches (RCMs) play a vital role in circRNAs biogenesis [28, 29], we hypothesized that RCMs may also regulate circPAPD4 synthesis. All possible RCMs were explored by investigating intron-flanking circPAPD4 sequence. We found two highly matched RCMs (81% identity over 598 nucleotides, Fig. S2), termed I1RCM (RCM in intron 1) and I4RCM (RCM in intron 4). We constructed overexpression plasmids containing pcDNA3.1 vector with linear sequences of exon 2 to exon 4 and RCMs (#1 wide type) inserted, and a series of deletions (#2-#4, deletion of I1RCM, I4RCM, and both RCMs) (Fig. 3A). After transfection with these vectors, the wide-type vector facilitated the expression of circPAPD4 compared to the other deletion vectors, indicating that I1RCM and I4RCM were responsible for circPAPD4 biogenesis in BC cells (Fig. 3B).

Recent studies reported that ADAR1 have promising therapy value as a potent circRNA regulator for a variety of cancers [29–32]. Although ADAR1 is an acknowledged proto-oncogene for BC, the underlying mechanism was largely unknown [29, 33–35]. Here, we assumed that ADAR1 could bind the flanking regions of circPAPD4 in BC cells. Our results demonstrated that knockdown of ADAR1 notably elevated circPAPD4 levels (Fig. 3C). Furthermore, anti-ADAR1 RNA immunoprecipitation (RIP) revealed substantial enrichment of I1RCM and I4RCM (Fig. 3D-E). Additionally, a negative correlation between circPAPD4 and ADAR1 expression was observed in BC tissues (Fig. 3F). Collectively, these results indicate that ADAR1 modulates circPAPD4 circularization by binding to its flanking intron complementary sequences.

### circPAPD4 serves as a sponge for miR-1269a to suppress BC progression

**Fig. 4:**
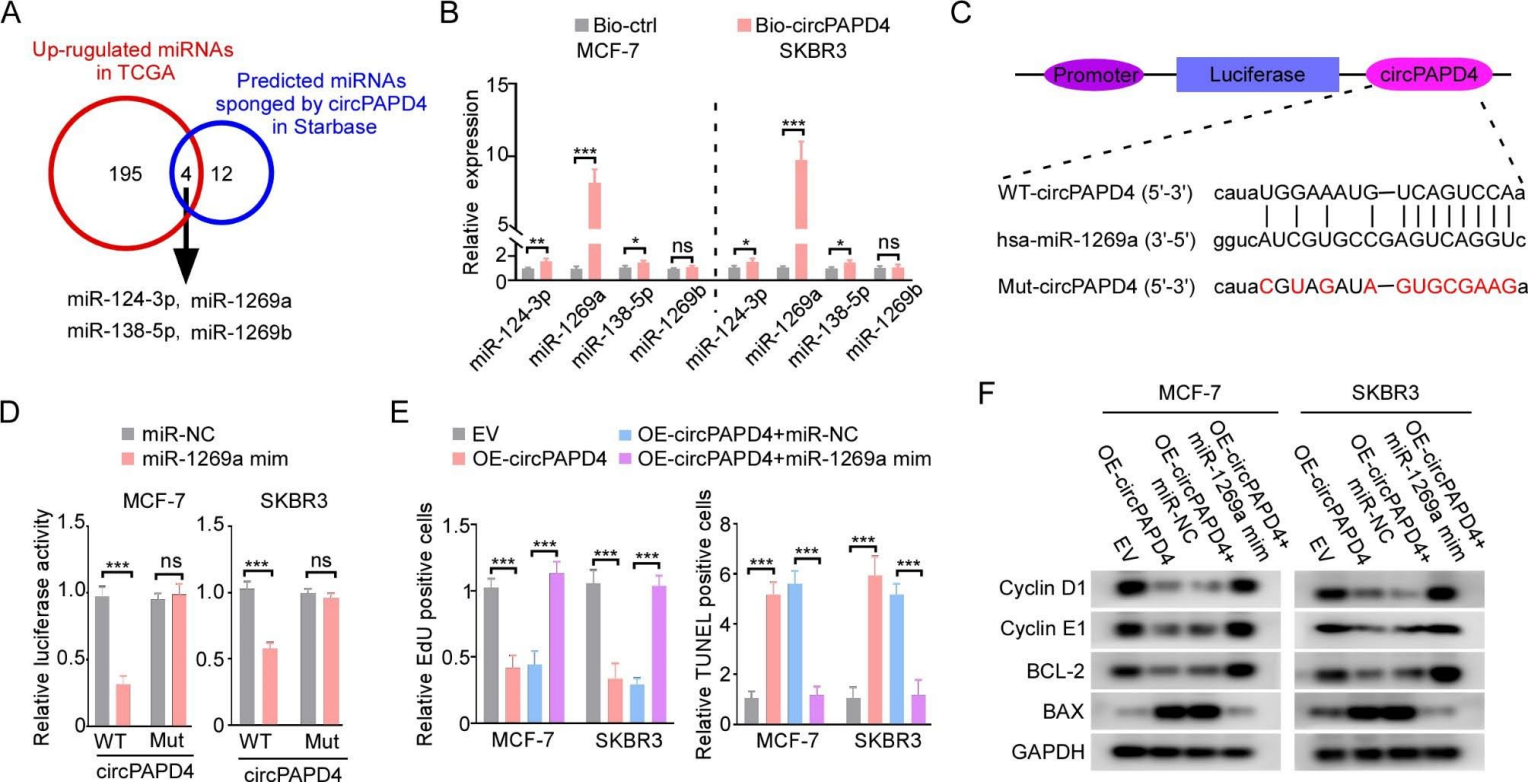
circPAPD4 inhibits growth and induces apoptosis of BC cells by sponging miR-1269a. **(A)** Venn diagram of potential sponged miRNAs of circPAPD4 in Starbase prediction and significantly up-regulated miRNAs in TCGA-BRCA. **(B)** After BC cells were treated with circPAPD4 specific probe (Bio-circPAPD4) or control probe (Bio-control), the enrichment of potential sponged miRNAs (miR-124-3p, miR-1269a, miR-138-5p, miR-1269b) were evaluated by RT-qPCR. **(C)** Schematic diagram demonstrated luciferase reporter vectors were inserted with wild-type (WT) or mutant (Mut) potential binding sequence between miR-1269a and circPAPD4. **(D)** Luciferase reporter vectors loaded with circPAPD4 wild-type (WT) or mutant (Mut) were co-transfected with miR-1269a mimics (miR-1269a mim) or mimics control (miR-NC), and then relative luciferase activity were assessed to verified the binding relationship between miR-1269a and circPAPD4 in BC cells. **(E)** The proliferation and apoptosis of MCF-7 and SKBR3 cells was measured by EdU and TUNEL assays after co-transfected with EV, OE-circPAPD4, OE-circPAPD4 + miR-NC, or OE-circPAPD4 + miR-1269a mim. **(F)** The expression of cyclin-D1, cyclin-E1, BCL-2, and BAX were evaluated by western blotting in MCF-7 and SKBR3 cells as indicated treatments. Data are presented as means ± SD. *p < 0.05; **p < 0.01; ***p < 0.001.

Given that circRNAs localized in the cytoplasm have been well acknowledged as being miRNA sponges [36, 37], we hypothesized that circPAPD4 exerts an anti-tumor role by functioning similarly. Therefore, we performed bioinformatics analysis using Starbase (http://starbase.sysu.edu.cn/) to identify the possible miRNA-binding partners of circPAPD4. Considering the low expression of circPAPD4 in BC tissues, we focused on differentially upregulated miRNAs in the TCGA database (https://www.tcga.org/) to explore candidate miRNAs. Subsequently, we constructed a Venn diagram to screen the specific miRNAs that may be bound by circPAPD4. Four miRNAs (miR-124-3p, miR-1269a, miR-138-5p, and miR-1269b) were found to possibly bind to circPAPD4 (Fig. 4A). Moreover, miR-1269a exhibited stronger specific enrichment in circPAPD4 pull-down pellets through RNA pull-down assays than the other miRNAs (Fig. 4B), and overexpression of miR-1269a in BC tissues was validated using RT-qPCR (Fig. S3). Therefore, we chose miR-1269a for subsequent studies. To further identify the binding sites between circPAPD4 and miR-1269a from Starbase (Fig. 4C), luciferase reporter assays demonstrated that relative luciferase activity was substantially decreased in the circPAPD4-WT group after co-transfection with miR-1269a mimics; however, no distinct change was observed in the circPAPD4-Mut group after co-transfection with miR-1269a mimics. This indicated that circPAPD4 could bind to miR-1269a (Fig. 4D). To further investigate whether miR-1269a could reverse the biological functions induced by circPAPD4 overexpression, rescue experiments were conducted with additional treatments of miR-1269a mimics based on circPAPD4-overexpressing BC cells. EdU staining revealed that miR-1269a mimics effectively rescued proliferation suppression induced by circPAPD4 overexpression in MCF-7 and SKBR3 cells (Fig. 4E). In addition, TUNEL analyses indicated that the apoptosis-promoting effect of circPAPD4 overexpression was blocked by miR-1269a mimics in MCF-7 and SKBR3 cells (Fig. 4E). We also observed that miR-1269a mimics restored the expression of cyclin D1, cyclin E1, and BCL-2, and decreased BAX expression in circPAPD4-overexpressing BC cells (Fig. 4F). Collectively, these data suggest that circPAPD4 overexpression suppresses breast cancer progression by acting as a sponge for miR-1269a.

### CircPAPD4 overexpression suppresses breast cancer progression by regulating miR-1269a/CREBZF axis

**Fig. 5:**
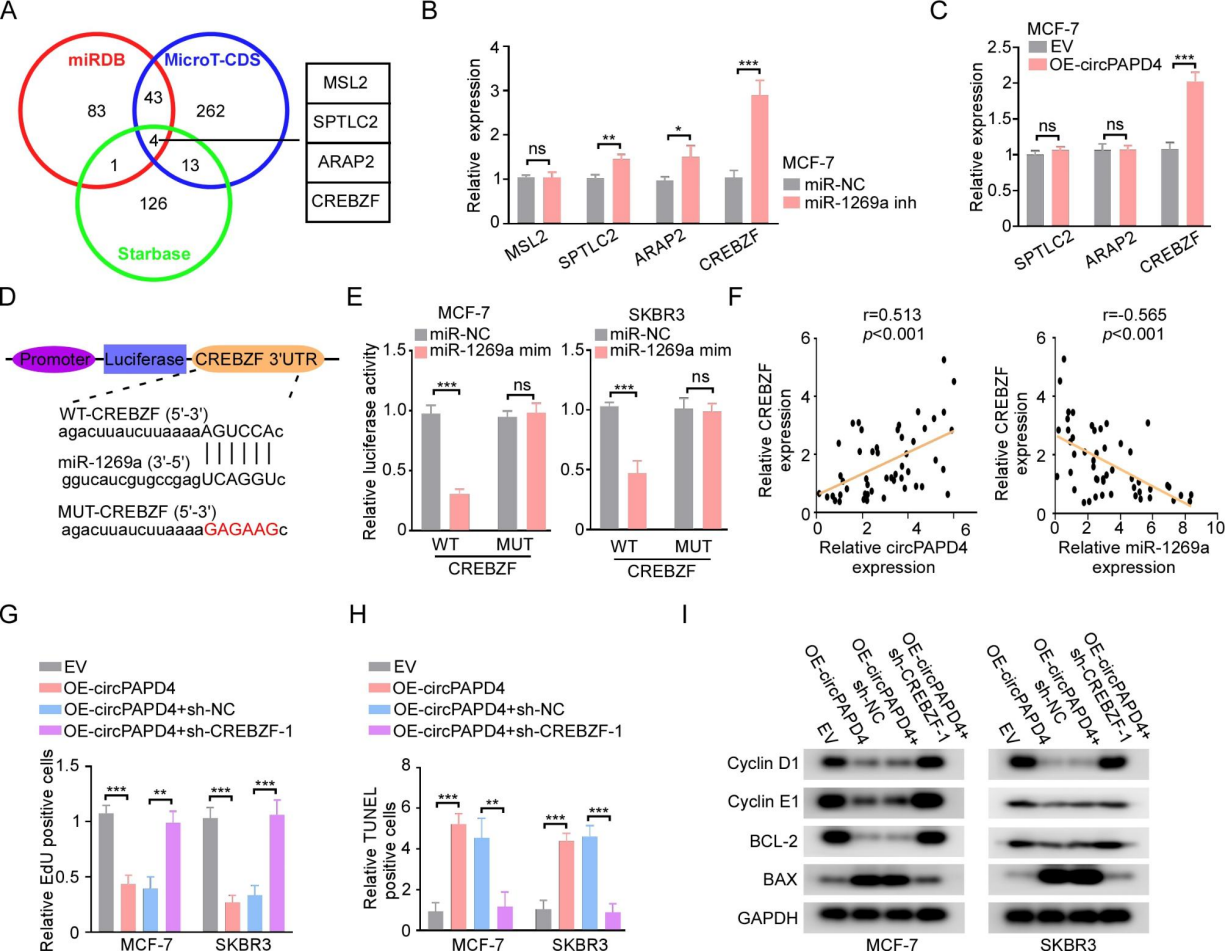
CREBZF is a directly target gene of circPAPD4/miR-1269a axis in BC cells. **(A)** Venn diagram of potential target genes of miR-1269a in Starbase, miRDB and microT-CDS. **(B)** mRNA expression levels of potential target genes (MSL2, SPTLC2, ARAP2, CREBZF) in MCF-7 cells transfected with miR-1269a inhibitors (miR-1269a inh) or miR-NC were detected by RT-qPCR. **(C)** The mRNA expression of SPTLC2, ARAP2, and CREBZF in MCF-7 cells transfected with OE-circPAPD4 or EV was detected by RT-qPCR. **(D)** Schematic diagram demonstrated luciferase reporter vectors were inserted with wild-type (WT) or mutant (Mut) potential binding sequence between miR-1269a and CREBZF which was predicted by Starbase. **(E)** Luciferase reporter vectors loaded with CREBZF wild-type (WT) or mutant (Mut) were co-transfected with miR-1269a mimics (miR-1269a mim) or mimics control (miR-NC), and then relative luciferase activity were assessed to verified the binding relationship between CREBZF and miR-1269a. **(F)** Pearson’s correlation analysis were used to analysis the correlation among CREBZF, miR-1269a, and circPAPD4 in 50 BC tissues. **(G-H)** The proliferation and apoptosis of MCF-7 and SKBR3 cells were measured by EdU and TUNEL assays after co-transfected with EV, OE-circPAPD4, OE-circPAPD4 + sh-NC, or OE-circPAPD4 + sh-CREBZF-1. **(I)** The levels of cyclin-D1, cyclin-E1, BCL-2, and BAX were evaluated by western blotting in MCF-7 and SKBR3 cells as indicated treatments. Data are presented as means ± SD. *p < 0.05; **p < 0.01; ***p < 0.001.

miRNAs affect the biological function of cells by modulating the expression of target genes [38, 39]. To determine the possible target genes of miR-1269a, we searched three datasets including Starbase, miRDB, and MicroT-CDS, and identified 4 overlapping potential genes (MSL2, SPTLC2, ARAP2, and CREBZF) (Fig. 5A). Among the 4 candidate genes, the mRNA expression of SPTLC2, ARAP2, and CREBZF was notably upregulated by the miR-1269a inhibitor in MCF-7 cells (Fig. 5B). Additionally, we also measured the changes of SPTLC2, ARAP2, and CREBZF after overexpressing circPAPD4. CREBZF mRNA expression was significantly enhanced in circPAPD4-overexpressing BC cells (Fig. 5C). Using the predicted sequence from Starbase, we constructed plasmid and mutant vectors, including CREBZF 3′-UTRs with wild-type and mutant sequences (Fig. 5D). Subsequently, the dual luciferase reporter assay results confirmed that CREBZF was the downstream target gene of miR-1269a (Fig. 5E). Consistently, we found that there was a positive relationship between circPAPD4 and CREBZF expression and a negative correlation between CREBZF and miR-1269a expression in BC tissues (Fig. 5F). Additionally, RT-qPCR verified that CREBZF was down-regulated in BC tissues compared to in their paracancerous tissues (Fig. S4A). Based on the median expression of CREBZF in BC tissues as measured using IHC, we divided the high- and low-expression groups to investigate the prognostic value of CREBZF (Fig. S4B). Low CREBZF expression was significantly correlated with poor RFS and could be served as an independent prognostic factor for RFS in BC (Fig. S4C, Table S8).

To further investigate whether circPAPD4 inhibits breast cancer progression through CREBZF, we silenced CREBZF expression using shRNA (sh-CREBZF) in MCF-7 and SKBR-3 cells (Fig. S4D). Then, we co-transfected circPAPD4 overexpression vector and CREBZF shRNA into MCF-7 and SKBR3 cells. Western blot results demonstrated that enhancement of CREBZF expression by circPAPD4 overexpression was successfully inhibited by CREBZF shRNA (Fig. S4E). In subsequent EdU staining and TUNEL analyses, the effects of circPAPD4 overexpression on the suppression of proliferation and promotion of apoptosis were reduced after CREBZF knockdown in MCF-7 and SKBR3 cells (Fig. 5G-H). Additionally, the downregulation of CREBZF restored the expression of cyclin D1, cyclin E1, and BCL-2 and decreased BAX expression in circPAPD4-overexpressing BC cells (Fig. 5I). In summary, our results indicate that circPAPD4 overexpression inhibits breast cancer progression by modulating the miR-1269a/CREBZF axis.

### CREBZF inhibits transcription of ADAR1 by suppressing dimerization of STAT3

**Fig. 6:**
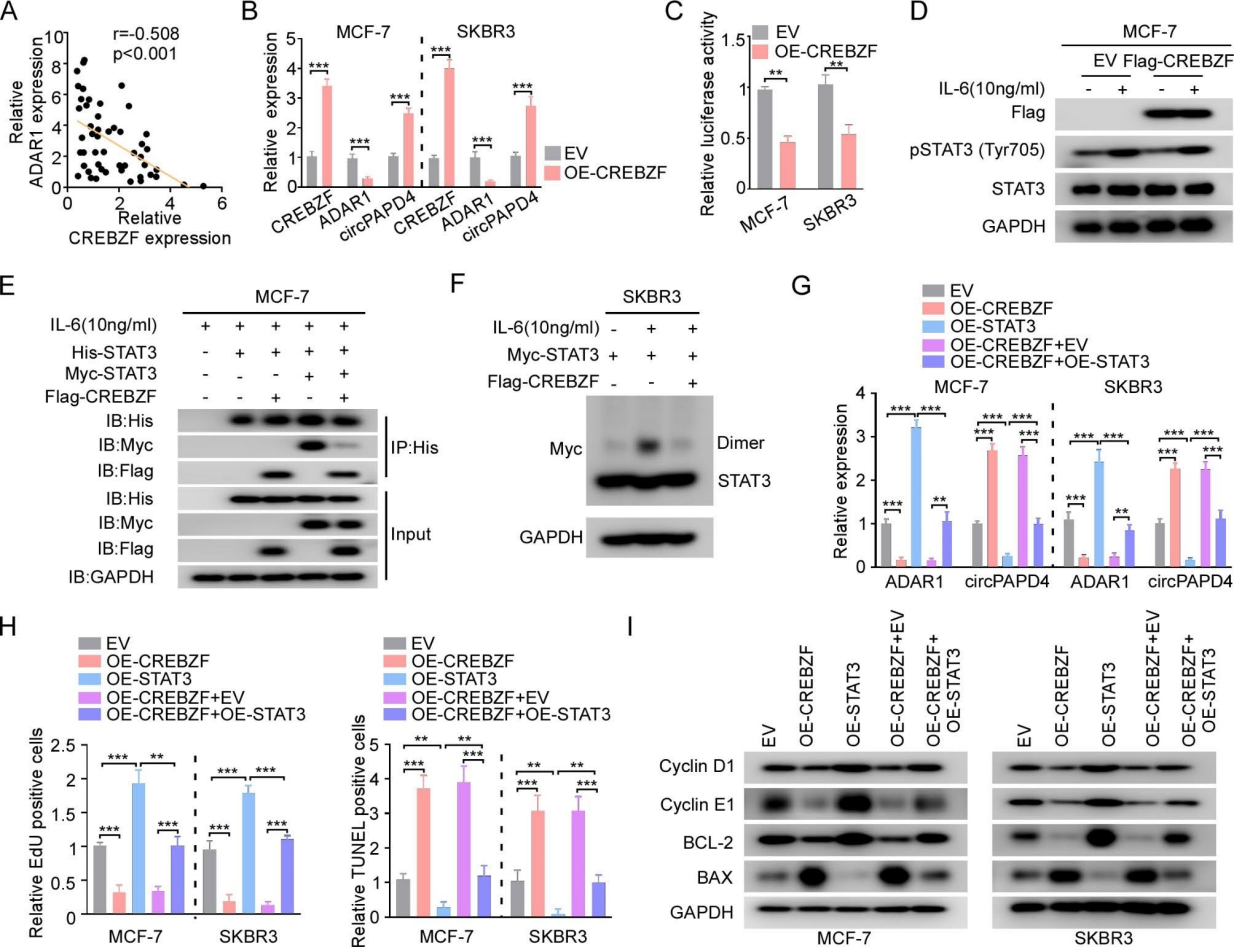
CREBZF suppresses ADAR1 expression via repressing STAT3 dimerization, leading to the biogenesis of circPAPD4. **(A)** Correlation between CREBZF and ADAR1 expression in 50 BC tissues were measured by Pearson’s Correlation analysis. **(B)** Relative expression of CREBZF, ADAR1, and circPAPD4 in MCF-7 and SKBR3 cells treated with OE-CREBZF or EV were assessed by RT-qPCR. **(C)** STAT3 luciferase reporter vectors were co-transfected with EV or OE-CREBZF into BC cells, and transcriptional activity of STAT3 were measured with relative luciferase activity. **(D)** After transfected with Flag-tagged CREBZF or EV, cells were treated with or without 10 ng/mL IL-6 stimulation. Then, immunoblots were performed to evaluate the expression levels of Flag, p-STAT3, and STAT3. **(E)** His-tagged STAT3 was co-transfected with Myc-tagged STAT3 and Flag-tagged CREBZF in the stimulation of 10 ng/mL human IL-6 into MCF-7 cells. Anti-His immunoprecipitates were immunoblotted with His, Myc, and Flag antibodies. **(F)** SKBR3 cells were co-transfected with Myc-STAT3 and Flag-CREBZF, followed by treatment with 10 ng/mL IL-6 stimulation; Then, STAT3 dimerization assay was conducted. **(G)** Relative RNA expression of ADAR1 and circPAPD4 in BC cells were detected by RT-qPCR after co-transfected with EV, OE-CREBZF, OE-STAT3, OE-CREBZF + EV, or OE-CREBZF + OE-STAT3. **(H)** The proliferation and apoptosis of MCF-7 and SKBR3 cells as indicated treatments were measured by EdU and TUNEL assays. **(I)** The expression of cyclin-D1, cyclin-E1, BCL-2, and BAX were evaluated by western blotting in MCF-7 and SKBR3 cells as indicated treatments. Data are presented as means ± SD. **p < 0.01; ***p < 0.001.

Notably, in the RT-qPCR analysis on clinical BC samples, we firstly found that CREBZF expression was negatively correlated with ADAR1 levels (Fig. 6A). And overexpression of CREBZF significantly downregulated ADAR1 expression and increased circPAPD4 levels (Fig. 6B), suggesting that CREBZF may regulate the transcription of ADAR1 and further involve in circPAPD4 biogenesis. It has been reported that CREBZF controlled liver regeneration via regulating the activity of STAT3 pathway [21]. Luciferase reporter assays revealed a distinct decrease in STAT3-responsive luciferase activity (4×M67 pTATA TK-Luc reporter) in CREBZF-overexpressing BC cells, indicating that CREBZF overexpression hindered STAT3 transcriptional activity in BC cells (Fig. 6C).

STAT3 is canonically activated by phosphorylation at Y705 upon stimulation with a variety of cytokines, and p-STAT3 Y705 dimerizes and migrates to the nucleus and furtherly induces the expression of target genes [40, 41]. As the phosphorylation of STAT3 was inconspicuously altered by CREBZF overexpression in MCF-7 cells (Fig. 6D), therefore we hypothesized that CREBZF might interfere with the dimerization of STAT3 to modulate its activity. As shown in Fig. 6E, MCF-7 cells were co-transfected with His-tagged STAT3, Myc-tagged STAT3, and Flag-tagged CREBZF plasmids. Co-immunoprecipitation assays confirmed the combination between these two STAT3 forms in the presence of IL-6. Notably, CREBZF overexpression strongly hindered STAT3 dimerization (Fig. 6E). The suppressive effects of CREBZF on STAT3 dimerization were verified in SKBR3 cells using a STAT3 dimerization assay (Fig. 6F). Notably, ADAR1 has been reported as a downstream gene of STAT3 [17]; therefore, we assumed that CREBZF regulates ADAR1 expression by modulating transcriptional activity of STAT3. RT-qPCR assays indicated that overexpression of CREBZF downregulated ADAR1 expression and increased circPAPD4 levels, whereas additional treatment with STAT3 overexpression reversed the regulatory effect of CREBZF (Fig. 6G). EdU staining and TUNEL assay results showed that the effects of CREBZF overexpression on suppressing proliferation and promoting apoptosis were restrained in upregulated STAT3 BC cells (Fig. 6H). Western blot results suggested that the up-regulation of CREBZF could suppress the expression of cyclin D1, cyclin E1, and BCL-2, as well as increase BAX expression; these effects were reversed by STAT3 overexpression (Fig. 6I). Collectively, these results indicate that CREBZF blocks STAT3 dimerization and further inhibits ADAR1 expression, thereby enhancing circPAPD4 expression. Thus, a novel positive feedback loop (circPAPD4/miR-1269a/CREBZF/STAT3/ADAR1) in BC was established.

### Functional NPs delivery of CREBZF mRNA to CREBZF-null breast cancer cells in vitro and pharmacokinetics and biodistribution of NPs in vivo

**Fig. 7:**
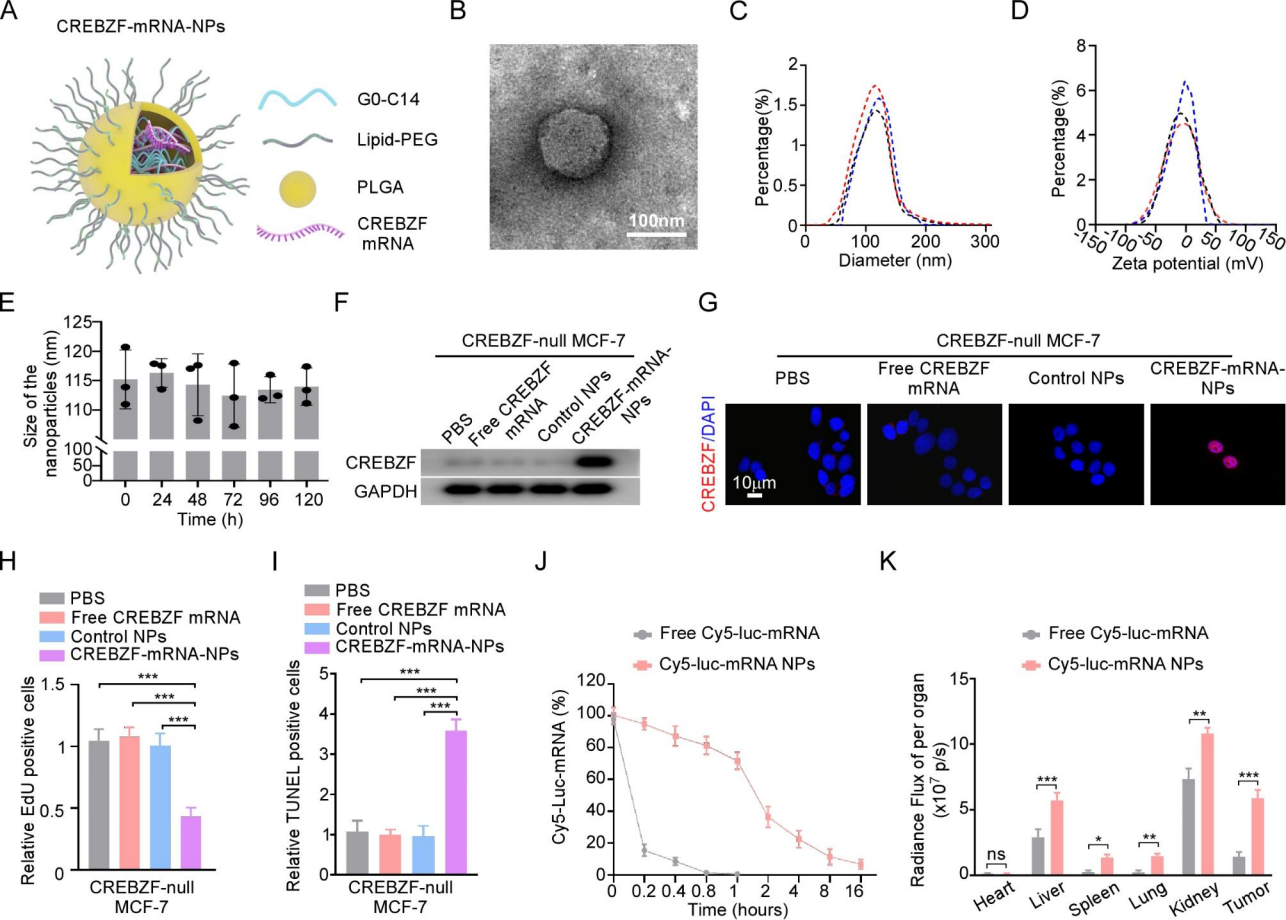
CREBZF restoration by CREBZF mRNA NPs (CREBZF-mRNA-NPs) exerts anti-tumor effect in vitro. **(A)** Schematic diagram illustrated that CREBZF-mRNA-NPs consisted of PLGA polymers, lipid-PEG, cationic G0-C14 and CREBZF mRNA. **(B)** Representative TEM image of CREBZF-mRNA-NPs. Scale bar: 100 nm. **(C-D)** The hydrodynamic size and zeta potential of CREBZF-mRNA-NPs was evaluated by dynamic light scattering (DLS). **(E)** Stability of the CREBZF-mRNA-NPs in 10% serum at 37 °C was measure by NanoSIGHT and NTA. **(F-G)** The restoration of CREBZF by CREBZF-mRNA-NPs was measured by western blotting **(F)** and immunofluorescence **(G)**. Scale bar: 10 μm. **(H-I)** The proliferation and apoptosis of CREBZF-null MCF-7 cells as indicated treatments were measured by EDU and TUNEL assays. **(J)** The half-life period of free Cy5-Luc-mRNA and Cy5-Luc-mRNA NPs in mice blood after i.v. administration. **(K)** Quantification of biodistribution of free Cy5-Luc-mRNA and Cy5-Luc-mRNA NPs in injection of CREBZF-null MCF-7 cells xenograft models of nude mice. Data are presented as means ± SD. *p < 0.05; **p < 0.01; ***p < 0.001.

Based on the core function of CREBZF in the circPAPD4/miR-1269a/CREBZF/STAT3/ADAR1 feedback loop, we established efficient NP-mediated mRNA delivery system to restore CREBZF expression in BC cells. Referring to previous studies [42], the core of the NPs was formed by PLGA polymers, and the cationic G0-C14 compound with CREBZF mRNA were encapsulated into NPs. Lipid-poly (ethylene glycol) (lipid-PEG) was used to build a shell for the NPs to improve their stability (Fig. 7A). Transmission electron microscopy (TEM) revealed the CREBZF-mRNA-NPs were spherical (Fig. 7B). Dynamic light scattering (DLS) indicated that the size of hybrid CREBZF-mRNA-NPs was about 115.9 nm (Fig. 7C) and the zeta potential was about − 8.1 mV (Fig. 7D). In addition, the stability of CREBZF-mRNA-NPs in the presence of 10% serum was confirmed over 120 h (Fig. 7E). To verify the restoration efficacy of CREBZF-mRNA-NPs, we constructed CREBZF knockout MCF-7 cells (CREBZF-null MCF-7) using CRISPR/Cas9 technology (Fig. S5) and checked CREBZF protein expression after treatment with PBS, free CREBZF mRNA, control NPs, and CREBZF-mRNA-NPs. Both western blotting and immunofluorescence staining demonstrated that CREBZF expression was successfully restored in CREBZF-null MCF-7 cells treated with CREBZF-mRNA-NPs (Fig. 7F-G). Functionally, CREBZF-mRNA-NPs treatment successfully inhibited growth and reversed apoptosis-inhibitory activity in CREBZF-null MCF-7 cells (Fig. 7H-I).

The efficiency of our mRNA-NPs delivery system in vivo was evaluated with pharmacokinetics (PK). After administering Cy5-luc-mRNA NPs or free Cy5-Luc-mRNA into healthy nude mice through tail vein, we observed that almost 95% of the free Cy5-luc-mRNA was degraded within 0.4 h. In contrast, approximately 20% of the Cy5-Luc-mRNA NPs remained detectable in circulation after 4 h, indicating that mRNA-NP delivery prolonged mRNA circulation (Fig. 7J). We accessed the biodistribution (BioD) and tumor accumulation of mRNA-NPs in nude mice with xenograft tumors. Tumor-bearing mice received free Cy5-Luc-mRNA or Cy5-Luc-mRNA NPs via tail vein injection. We observed that high NPs accumulated in kidney and liver after i.v. administration. More importantly, Cy5-luc-mRNA NPs exhibited high tumor accumulation in the MCF-7 xenograft, whereas barely no signals in tumor were detected for free Cy5-luc-mRNA (Fig. 7K). Collectively, these findings indicate that CREBZF-mRNA-NPs could effectively deliver CREBZF mRNA to tumor cells, reduce proliferation, and promote apoptosis in CREBZF-null MCF-7 cells.

### Therapeutic efficacy and toxicity of systemic CREBZF-mRNA-NPs administration of in breast cancer xenograft

**Fig. 8:**
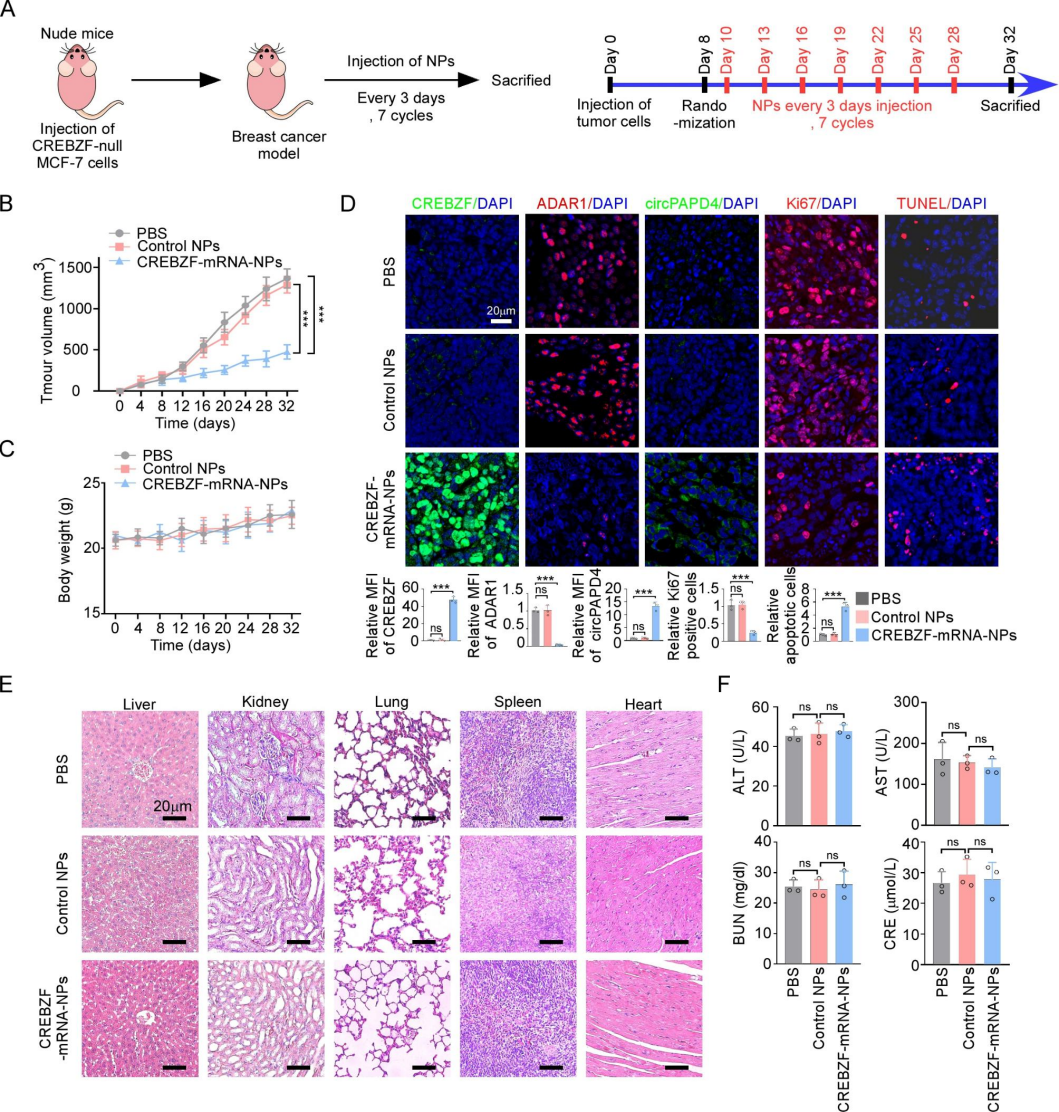
The therapeutic efficacy and toxicity of CREBZF-mRNA-NPs for BC in vivo. **(A)** Schematic diagram illustrated the timeline of tumor planted and injection of PBS, Control NPs, or CREBZF-mRNA-NPs in CREBZF-null MCF-7 nude mice. After 8 days of tumor inoculation, mice received NPs injection every 3 days for 7 cycles. **(B)** Tumor growth curves of CREBZF-null MCF-7 xenograft mice after randomly injection of PBS, Control NPs, or CREBZF-mRNA-NPs group. **(C)** Body weight of nude mice in indicated group. **(D)** CREBZF, ADAR1, circPAPD4, Ki-67, and TUNEL expression levels of xenografts in indicated groups were evaluated by immunofluorescence and FISH analysis; Scale bar: 20 μm. **(E)** Represented H&E stained images of major organs after treated with PBS, Control NPs, or CREBZF-mRNA-NPs; Scale bar: 20 μm. **(F)** Serum levels of ALT, AST, Cr, and BUN in indicated groups after 24 h from i.v. injection. n = 3 mice each group. Data are presented as means ± SD. ***p < 0.001.

To verify the therapeutic feasibility of CREBZF-mRNA-NPs in breast cancer xenograft model, we systemically injected CREBZF-mRNA-NPs through tail vein in nude mice bearing CREBZF-null MCF-7 xenograft tumors every three days for seven injections cycles. CREBZF-mRNA-NPs considerably suppressed tumor growth, while both control groups (treatment with PBS and control NPs) showed rapid tumor growth (Fig. 8A-B). Body weights of mice in each group did not differ significantly (Fig. 8C). In addition, compared to either PBS or control NPs, immunofluorescence and FISH analysis of tumor sections revealed that CREBZF-mRNA-NPs promoted CREBZF and circPAPD4 expression, inhibited ADAR1 expression, and significantly discouraged proliferation while encouraging apoptosis of tumor cells (Fig. 8D). Representative H&E staining showed that CREBZF-mRNA-NPs injection was not toxic to major organs (Fig. 8E). In addition, blood index analyses of ALT, AST, Cr, and BUN levels showed no significant hepatotoxicity or renotoxicity (Fig. 8F). Collectively, these results indicate that CREBZF-mRNA-NPs effectively suppressed tumor progression with negligible side effects.

## Discussion

Owing to the evolution of second-generation sequencing, the oncogenic role of dysregulated circRNAs in multiple cancers has drawn significant scientific attention [43]. Given the high stability of circRNAs along with their tissue-specific expression, they may be potential novel biomarkers for BC diagnosis and prognosis [44]. However, the detailed mechanism of how they impact BC development and progression remains unclear. Based on the data from the GEO database and the results of our assays, we identified a novel circRNA, circPAPD4, which was substantially downregulated in BC tissues and cells, suggesting that it might play an anti-oncogenic impact on BC progression. CircPAPD4 expression was negatively associated with the advanced stages of BC, and patients with lower circPAPD4 expression had significantly shorter RFS. Functionally, up-regulated circPAPD4 slowed proliferation and promoted apoptosis of BC cells both in vitro and in vivo.

Most circRNAs consist of several exons from the pre-mRNA of genes, and introns are removed during their generation. Notably, complementary repeating sequences are found on the introns flanking the back-spliced exons; several studies have demonstrated that RNA binding proteins (RBPs) bind to these sequences and modulate exon circularization [15, 45]. ADAR1, an adenosine deaminase, is characterized as a catalysator of adenosine-to-inosine (A-to-I) RNA editing [46]. Recent reports revealed that ADAR1 decreases several circRNAs biogenesis via reducing the complementarity of RCM sequences [13, 14]. In addition, some studies have demonstrated that ADAR1 is overexpressed and acts as a cancer-promoting gene in BC [33, 47]; however, the precise molecular mechanism behind this effect remains unknown. In the present study, we confirmed that ADAR1 binds to the RCM of flanking introns and suppresses the generation of circPAPD4. Consistently, ADAR1 mRNA expression negatively correlated with circPAPD4 expression in clinical tissues. These results revealed a novel mechanism where ADAR1 promotes BC progression by inhibiting circPAPD4 biogenesis.

Accumulating evidence has revealed various circRNAs functions, such as modulating the expression of parental genes through specific RNA-RNA interactions [48], acting as natural miRNA sponges (ceRNA) [49], and assembling protein complexes [50]. Among these, ceRNA are the most predominant. Numerous studies have demonstrated that circRNAs regulate the expression of target genes through miRNAs interactions and influence the malignancy and progression of diverse cancers [45, 51, 52]. We found that circPAPD4 was predominantly enriched in the cytoplasm of BC cells; miRNA-binding prediction revealed binding sites for miR-1269a in circPAPD4, and we verified that circPAPD4 was capable of binding to miR-1269a via RNA pull-down and luciferase reporter assays. In recent years, miR-1269a has been reported to exert tumor-promoting effects in several types of cancers [53–55]. High expression of miR-1269a was found in colorectal cancer (CRC) tissues, and forced expression of miR-1269a significantly enhances metastasis of CRC cells, by forming a positive feedback loop with TGF-β [53]. Zhang et al. reported that miR-1269a promotes proliferation and suppresses apoptosis of glioma cells by directly regulating ATRX [56]. Overexpression of LncRNA LINC00261 inhibits the growth and metastasis of lung cancer by modulating the miR-1269a/FOXO1 axis [54], in which miR-1269a serves as a carcinogenic miRNA. In our study, miR-1269a attenuated the circPAPD4-mediated effects on BC cell proliferation and apoptosis, showing circPAPD4 represses malignant properties of BC by competitively biding miR-1269a.

CREBZF is a leucine zipper (B-zip) protein. Instead of directly binding b-Zip response elements to fulfill its function, CREBZF activates transcription by interacting with other B-zip proteins, like activating transcription factor 4 (ATF4) [57, 58]. As previously reported, CREBZF may be associated with p53, ERK, apoptosis, and autophagy [19, 59]. Bodnarchuk et al. revealed that CREBZF induced cell death enhanced staining for autophagic vesicles, and promoted an autophagy response through gene expression in medulloblastoma cells [19]; however, the underlying modulatory mechanism behind this effect in BC cells remains unelucidated. Using bioinformatic prediction and dual-luciferase reporter assays, we demonstrated that miR-1269a was able to directly bind to the 3′ UTR of CREBZF. Additionally, we found that circPAPD4 regulates CREBZF expression. The correlation between circPAPD4, miR-1269a, and CREBZF was further verified in clinical tissues. Subsequently, we demonstrated that down-regulated CREBZF expression halted the circPAPD4 overexpression suppressing proliferation and promoting apoptosis in BC cells. Notably, CREBZF curbs cell cycle progression via inhibiting the dimerization and activation of STAT3 [21]. And STAT3-ADAR1 interplay drives the progression of multiple myeloma [17]. It is curious whether there is a closely correlation between CREBZF and STAT3/ADAR1 in BC. In present study, we firstly found that CREBZF was negatively correlated with ADAR1 in BC tissues and cell lines. Subsequent studies demonstrated that overexpression of CREBZF reduced ADAR1 expression via reducing the dimerization of STAT3, and finally elevated circPAPD4 expression, which eventually suppressed BC progression. Collectively, these results revealed a novel positive feedback loop in BC cells consisting of circPAPD4, miR-1269a, CREBZF, STAT3, and ADAR1.

The regulation of cell biological processes in normal and cancer cells requires the proper intracellular localization and nuclear-cytoplasm shuttling of specific proteins [60–62]. For example, p53, a protein that shuttles between the nucleus and cytoplasm, is normally expressed at low levels and found in both locations in unstressed cells. However, in response to DNA damage and other stressors, p53 undergoes post-translational modifications that stabilize and accumulate it in the nucleus, where it is activated as a transcription factor [63, 64]. Our study demonstrated that mature circPAPD4 is mainly located in the cytoplasm, while ADAR1, which localizes to the nucleus, binds to PAPD4 pre-mRNA, resulting in reduced circPAPD4 formation. The cytoplasmic circPAPD4 acts as a sponge for miR-1269a to enhance CREBZF expression. Previous studies have shown that CREBZF is present in both the nucleus and cytoplasm of osteosarcoma cells and human livers [65, 66]. Moreover, our study revealed that CREBZF inhibited the STAT3 pathway activation by preventing STAT3-STAT3 dimerization, rather than phosphorylation of STAT3. We hypothesized that CRZBZF located in both the cytoplasm and nucleus can suppress STAT3 dimerization, leading to significant inhibition of BCL2, Cyclin D1, Cyclin E1, and ADAR1 expression while inducing BAX level. Ultimately, the downregulation of ADAR1 accelerates circPAPD4 formation. Our findings provide further insight into the role of protein and nucleic acid cytoplasmic-nuclear transport in BC cells biological processes.

There are two isoforms of ADAR1: a longer isoform (ADAR1-p150) that can be induced by IFN and is present in both the cytoplasm and nucleus, and a shorter isoform (ADAR1-P110) that is typically found in the nucleus and expressed continuously [67, 68]. ADAR1 has been identified as a strong regulator of the circRNA transcriptome in various diseases [29, 69]. For example, Shi et al. reported that androgen receptor could suppress circRNA expression by upregulating ADAR1 p110 in hepatocellular carcinoma [32]. It has also been shown that ADAR1 p110 contributed to circRNA production suppression in gastric cancer [31]. In summary, we hypothesize that ADAR1 p110 is the primary factor responsible for the formation of circPAPD4 in breast cancer, while the involvement of ADAR1 p150 cannot be ruled out.

Over the past decade, gene therapy has attracted considerable attention to treat various diseases [70, 71]. Considering the potential genontoxicity, DNA therapies have not been widely accepted in clinical practice. Unlike DNA transfection, mRNA is unstable and degrades shortly after completing its specific functions in the cytoplasm. As it is safer, mRNA technologies show promise for effective in vivo regulation. Several studies have demonstrated tangible mRNA therapies, such as the regeneration of VEGF-A protein for diabetes [72], restoration of tumor suppressors for cancer treatment [73, 74], delivery of ARG1 mRNA liver-targeted NPs for arginase deficiency [23], and mRNA vaccines for COVID-19 by Moderna, Inc[22]. Nevertheless, the effective and safe systemic delivery of mRNA for target protein expression in BC cells remains challenge. In recent years, nanotechnology has widely shown promise in treatment for various diseases, and some NPs have been considered effective mRNA delivery agents for different RNA therapeutics [73–75]. In the present study, we constructed a polymeric NP platform for steadily delivering CREBZF mRNA to BC cells in vivo, where CREBZF-mRNA-NPs successfully curbed proliferation and enhanced apoptosis of BC tumors by activating the positive feedback loop of CREBZF/STAT3/ADAR1/circPAPD4/miR-1269a. Additionally, CREBZF-mRNA-NPs were specifically concentrated in tumors and no apparent damage or toxicity was observed in the major organs of mice. These results suggest that the restoration of CREBZF expression by NPs is a potentially safe and effective treatment strategy for BC.

## Conclusion

**Fig. 9:**
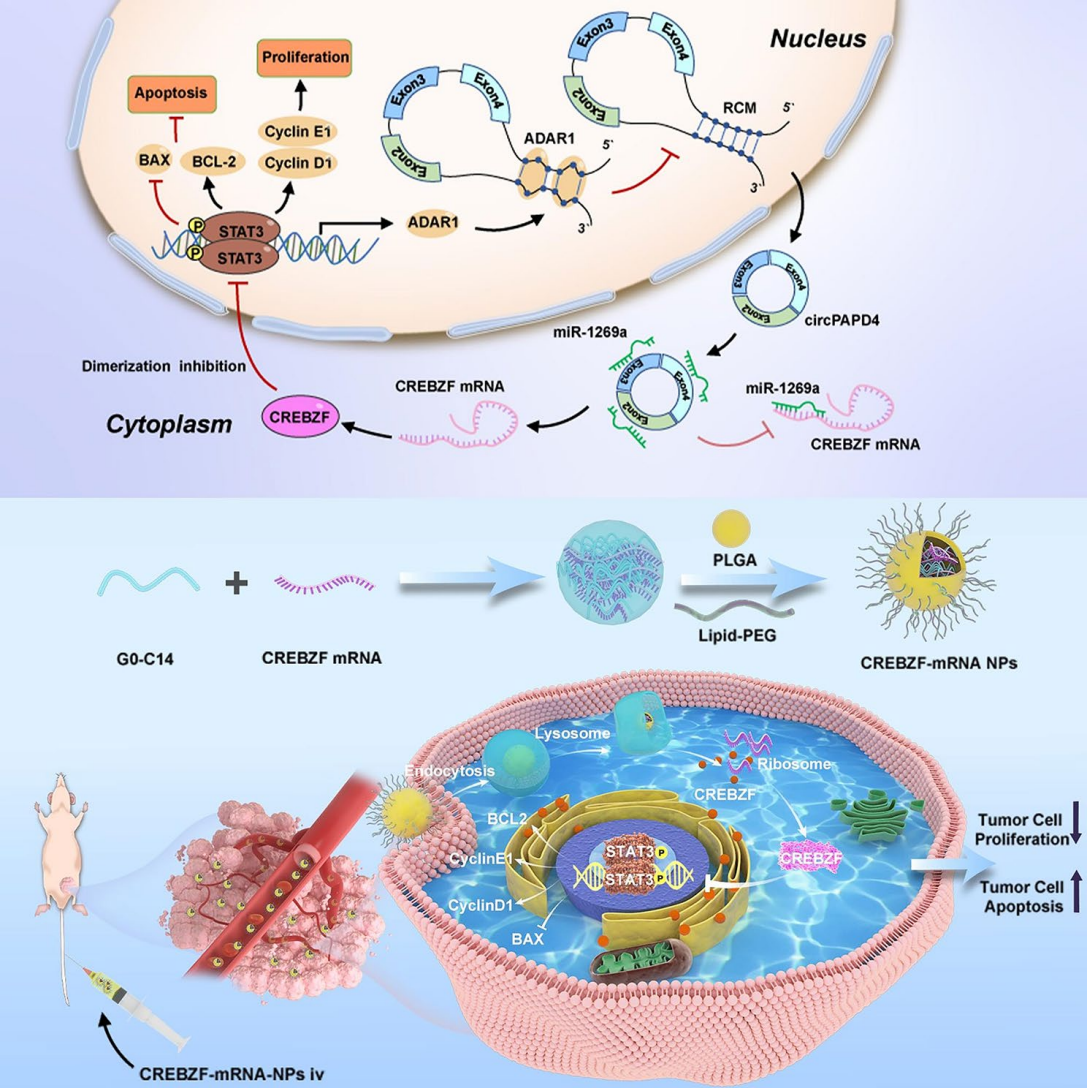
Function and mechanism of circPAPD4 in BC progression. Schematic diagram illustrates the molecular mechanism of circPAPD4 to curb BC progression via circPAPD4/miR-1269a/CREBZF/STAT3/ADAR1 positive feed-back loop. Moreover, NPs-medicated CREBZF effectively stimulates STAT3/ADAR1/circPAPD4/miR-1269a loops to restrain BC progression

In this study, we identified a new circRNA circPAPD4 that had decreased expression in both BC tissues and cell lines, and was negatively correlated with advanced cancer stages and poor prognosis. Furthermore, circPAPD4 exerted a sponge-like effect by binding miR-1269a and enhanced the expression of CREBZF, leading to the inhibition of STAT3 dimerization and ADAR1 expression, facilitating its biogenesis. Activation of this positive feedback loop by CREBZF-mRNA-NPs resulted in inhibited BC cell proliferation and promoted apoptosis. The circPAPD4/miR-1269a/CREBZF/STAT3/ADAR1 positive feedback loop provides novel insights into therapeutic strategies for BC (Fig. 9).

## Electronic supplementary material

Below is the link to the electronic supplementary material.

Supplementary Material 1

Supplementary Material 2

Supplementary Material 3

Supplementary Material 4

Supplementary Material 5

Supplementary Material 6

## Data Availability

The data sets used and/or analyzed during the current study are available from the corresponding author on reasonable request.

## Abbreviations

BC: Breast cancer
NPs: Nanoparticles
circRNAs: Circular RNAs
PAPD4: PAP-Associated Domain-Containing Protein 4
CREBZF: CREB/ATF BZIP Transcription Factor
ADAR1: Adenosine deaminase acting on RNA 1
STAT3: Signal transducer and activator of transcription 3
RT-qPCR: Real time quantitative polymerase chain reaction
ISH: in situ hybridization
IHC: Immunohistochemistry
CCK8: Cell Counting Kit-8
EdU: 5-Ethynyl-2’-deoxyuridine
TUNEL: terminal deoxynucleotidyl transferase dUTP nick end labeling
RFS: Recurrence-free survival
FISH: Fluorescent in situ hybridization
RCMs: reverse complementary matches
RIP: RNA immunoprecipitation
IHC: Immunohistochemistry
TEM: Transmission electron microscopy
PLGA: Poly lactic-co-glycolic acid
DLS: Dynamic light scattering
HCC: Hepatocellular carcinoma
H&E: Hematoxylin-eosin
ALT: Alanine transaminase
AST: Aspartate transaminase
Cr: Creatinine
BUN: Blood urea nitrogen
