# Correction: CREBZF mRNA nanoparticles suppress breast cancer progression through a positive feedback loop boosted by circPAPD4

**DOI:** 10.1186/s13046-023-02738-6

**Published:** 2023-07-04

**Authors:** Boxuan Zhou, Jinhua Xue, Runxin Wu, Hongyu Meng, Ruixi Li, Zhaohong Mo, Hang Zhai, Xianyu Chen, Rongqiang Liu, Guie Lai, Xiaohong Chen, Taiyuan Li, Shiyang Zheng

**Affiliations:** 1 Department of General Surgery, The First Affiliated Hospital of Nanchang University, Nanchang, 330000 China; 2 Department of Breast Surgery, The First Affiliated Hospital of Gannan Medical University, Gannan Medical University, Ganzhou, 341000 China; 3 Department of Physiology, School of Basic Medical Sciences, Gannan Medical University, Ganzhou, 341000 China; 4 Zhongshan School of Medicine, Sun Yat-Sen University, Guangzhou, 510080 China; 5 Department of Hepatobiliary Surgery, The Third Affiliated Hospital, Sun Yat-Sen University, Guangzhou, 510630 China; 6 Department of Hepatobiliary and Pancreatic Surgery, The Eighth Affiliated Hospital, Sun Yat-Sen University, Shenzhen, 518033 China; 7 Department of Hepatobiliary Surgery, Renmin Hospital of Wuhan University, Wuhan, 430060 China; 8 Department of Laboratory, The First Affiliated Hospital of Gannan Medical University, Ganzhou, 341000 China; 9 Department of Head and Neck Surgery, Cancer Center of Guangzhou Medical University, Guangzhou, 510060 China

**Correction:** ***J Exp Clin Cancer Res*** **42, 138 (2023)**

**https://doi.org/10.1186/s13046-023-02701-5**

Following publication of the original article [1], an error was identified in the author list. Authors, Boxuan Zhou, Jinhua Xue, Runxin Wu, Hongyu Meng, and Ruixi Li were not recognized as equal contributors and co-first authors.

The correction do not affect the overall Conclusion of the article. The original article has been corrected.

## References

[1] Zhou B, Xue J, Wu R (2023). CREBZF mRNA nanoparticles suppress breast cancer progression through a positive feedback loop boosted by circPAPD4. J Exp Clin Cancer Res.

