# Supplementary figures and images for: CREBZF mRNA nanoparticles suppress breast cancer progression through a positive feedback loop boosted by circPAPD4
Source: J Exp Clin Cancer Res. 2023 Jun 1;42:138. doi: 10.1186/s13046-023-02701-5 (PMC10233212; doi:10.1186/s13046-023-02701-5)

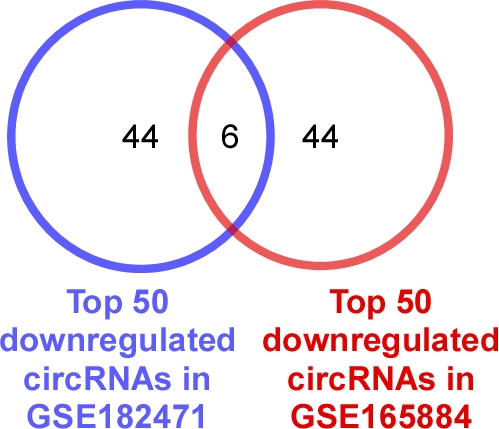

Supplement: Supplementary file 1 — Supplementary Material 1 [file 13046_2023_2701_MOESM1_ESM.jpg]

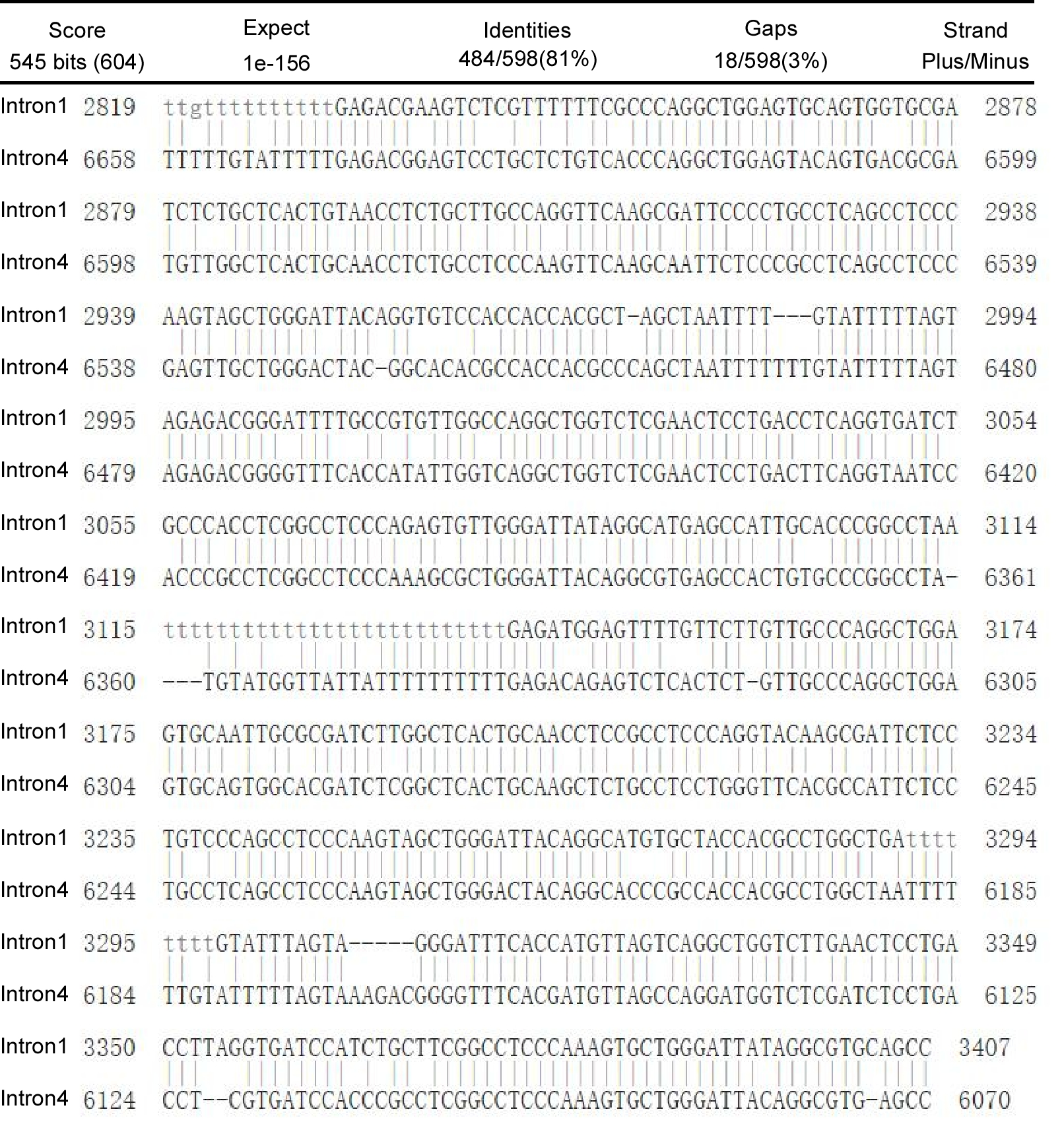

Supplement: Supplementary file 2 — Supplementary Material 2 [file 13046_2023_2701_MOESM2_ESM.jpg]

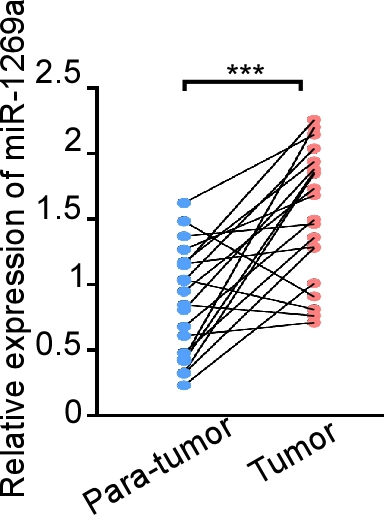

Supplement: Supplementary file 3 — Supplementary Material 3 [file 13046_2023_2701_MOESM3_ESM.jpg]

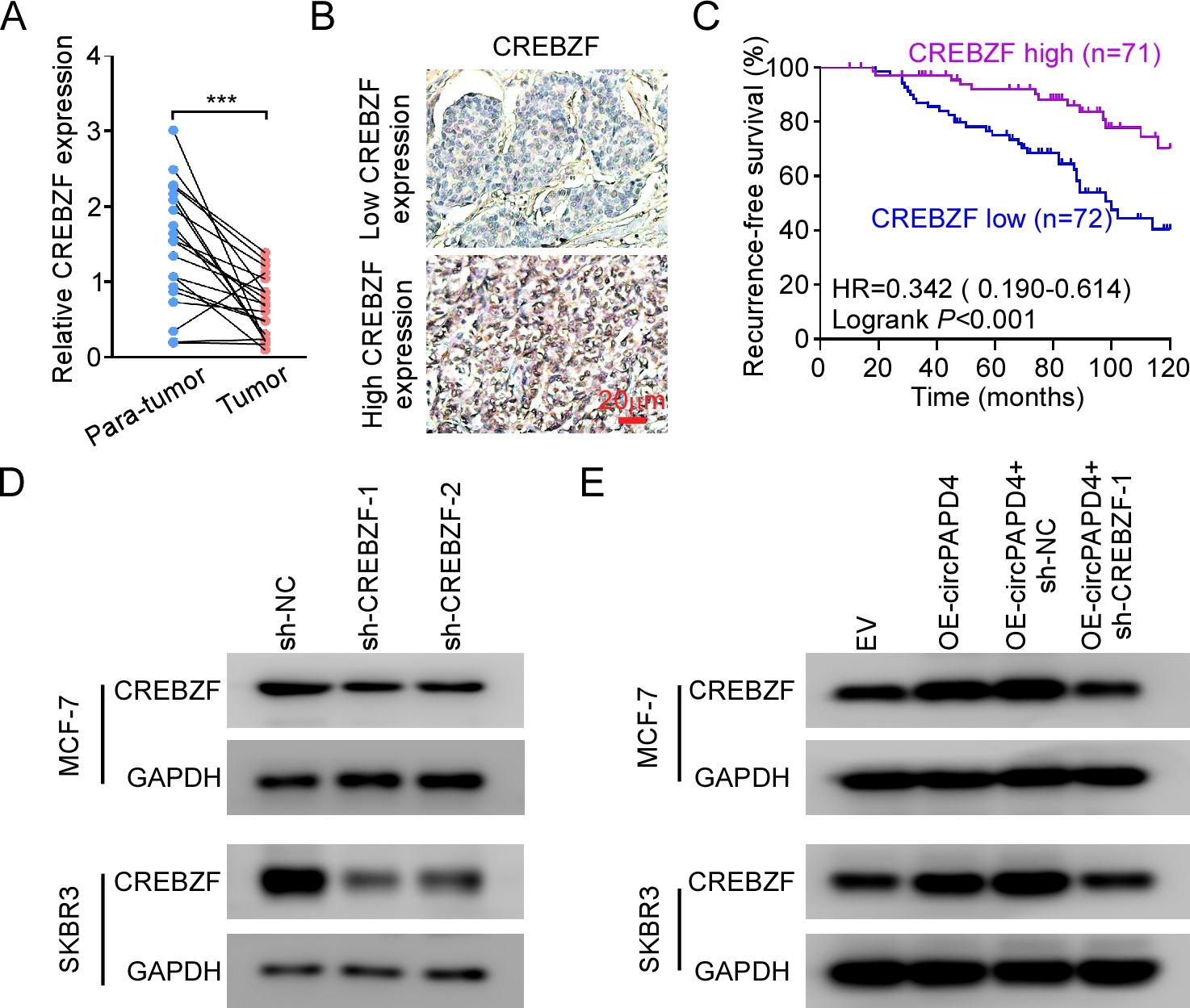

Supplement: Supplementary file 4 — Supplementary Material 4 [file 13046_2023_2701_MOESM4_ESM.jpg]

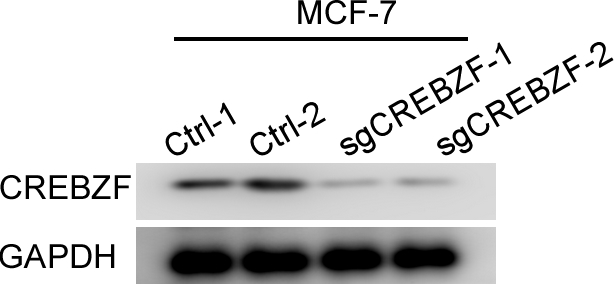

Supplement: Supplementary file 5 — Supplementary Material 5 [file 13046_2023_2701_MOESM5_ESM.jpg]
