# Supplementary material for: CREBZF mRNA nanoparticles suppress breast cancer progression through a positive feedback loop boosted by circPAPD4
Source: J Exp Clin Cancer Res. 2023 Jun 1;42:138. doi: 10.1186/s13046-023-02701-5 (PMC10233212; doi:10.1186/s13046-023-02701-5)
Supplement: Supplementary file 6 — Supplementary Material 6 [file 13046_2023_2701_MOESM6_ESM.docx]

**SUPPLEMENTAL MATERIALS**

**CREBZF mRNA nanoparticles suppress breast cancer progression by a positive feedback loop boosted by circPAPD4**

**Contents:**

1. Supplementary Experimental Procedures
2. Supplementary Table S1
3. Supplementary Table S2
4. Supplementary Table S3
5. Supplementary Table S4
6. Supplementary Table S5
7. Supplementary Table S6
8. Supplementary Table S7
9. Supplementary Table S8
10. Supplementary Table S9
11. Supplementary Table S10
12. Supplementary Figure legend

**Supplementary Experimental Procedures**

**RNA isolation and real-time quantitative polymerase chain reaction (RT-qPCR)**

The RNA extraction and RT-qPCR were carried out following previously described [1]. In brief, total RNA was extracted using TRIzol reagent (Invitrogen, USA) and reverse-transcribed into cDNA using a Reverse Transcription Kit (TakaRa, Japan) with the PrimeScript RT Reagent Kit and stem-loop primers. Subsequently, RT-qPCR was conducted using the SYBR Green Master Mix (TakaRa, Japan) on a LightCycler 480 (Roche, Switzerland) according to the manufacturer's instructions. The 2^-ΔΔCt^ method was employed to determine the relative RNA expression, and the primers used are listed in supplementary file Table S9.

**RNase R assay, Actinomycin D assay and Subcellular fractionation**

The RNase R assay involved treating 2 μg total RNA with 5U RNase R (Epicenter, USA) for 30 min at 37℃ [2], followed by RT-qPCR to measure the expression levels of circPAPD4 and PAPD4 mRNA.

To perform actinomycin D assay, breast cancer cells were seeded in 6-well plates at a density of 2×10^5^ cells/well and further incubated with 2 μg/ml Actinomycin D [3]. The cells were then collected at 0, 4, 8, 12 and 24h and the relative expression levels of circPAPD4 and PAPD4 mRNA were analyzed by RT-qPCR and normalized to the expression levels of the 0-hour group.

The cytoplasmic and nuclear RNA of breast cancer cells were isolated using cytoplasmic & Nuclear RNA purification Kit (Norgen Bioteck, Canada) as previously described [4]. The expression of nucleus control (U6), cytoplasm control (GAPDH), circPAPD4, and PAPD4 mRNA were analyzed individually by RT-qPCR.

**Fluorescence in situ hybridization (FISH) assay**

To perform the FISH assay, we firstly seeded breast cancer cells onto sterile glass slides and fixed them in 4% paraformaldehyde at room temperature (RT) for 15 mins. The cells were then permeabilized using PBS containing 0.05% Triton X-100 at 4℃ for 5 mins. The cells were next treated with prehybridization solution at 52℃ for 2 hours, followed by hybridization with anti-circPAPD4 oligodeoxynucleotide probe conjugated with DIG at 52℃ for 16 hours in hybridization solution. After washing in 2×SSC for 5 mins and 50% deionized formamide/4×SSC for 25 mins, we incubated the cells overnight at 4℃ with fluorescein-conjugated antibodies against DIG, and counterstained the nuclei with Hoechst 33342. Finally, we captured images using Laser scanning confocal microscopy (LSM800, Zeiss).

**In-situ hybridization (ISH) and Immunohistochemistry (IHC)**

To investigate the expression pattern of circPAPD4 in BC paraffin sections, we conducted ISH assay using the circRNA Hybridization Kit (Foco, China) and followed the manufactory’s protocol. Briefly, tumor tissue slides were first dewaxed, and then subjected to prehybridization solution before being hybridized with the circPAPD4 probe.

Standard procedures were followed for conducting Immunohistochemistry (IHC). Initially, the slides were deparaffinized using xylene and rehydrated with alcohol. Then, antigens were retrieved by placing the slides in a pressure cooker with 0.01 M citrate buffer or EDTA buffer for 3 mins. Subsequently, diluted hydrogen peroxide (3%) was used to inactivate endogenous peroxidase activity, followed by washing with phosphate-buffered saline (PBS). The sections were then sealed with 5% bovine serum albumin (BSA, YESEN, Shanghai, China) and incubated overnight at 4°C with anti-CREBZF (Abcam, USA) to determine the corresponding protein expression. The next day, the sections were incubated with a horseradish peroxidase (HRP)-labeled secondary antibody (Gene Tech, China) for 1 hour and then stained with diaminobenzidine (DAB, Gene Tech).

We used an Olympus microscope (Tokyo, Japan) to capture images of the slides and determined the expression level using IHC scores. The IHC scores were calculated by multiplying the staining intensity and the percentage of stained cells. Staining intensity was evaluated on the basis of the following degrees: 0, negative; 1, weak; 2, moderate; 3, strong. Proportion of stained cells was recorded on a scale of 1 (≤25%), 2 (25%-50%), 3 (50%-75%), 4 (>75%). To conduct survival analysis, we grouped patients based on the median expression of circPAPD4 and CREBZF, respectively.

**Immunofluorescence**

To fix the tumor tissue samples, we used 4% paraformaldehyde. The samples were then embedded in paraffin, sectioned, deparaffinized, and rehydrated. Antigens were retrieved using a pressure cooker for 3 mins in either 0.01 M citrate buffer or EDTA buffer. The sections were sealed with 5% BSA for 30 minutes before being incubated with anti-CREBZF antibody (Abcam, UK), anti-ADAR1 antibody (Abcam, UK), and anti-Ki-67 antibody (Abcam, UK) overnight at 4°C. After incubation with Alexa Fluor-conjugated secondary antibodies, we used Laser scanning confocal microscopy (LSM800, Zeiss) to capture images.

**Western blotting and Co-immunoprecipitation assay (Co-IP)**

We performed western blotting following the previously described [5]. In brief, we extracted protein lysates using RIPA lysis buffer (Beyotime, China), separated them on 10% SDS-polyacrylamide gels, and transferred them to polyvinylidene difluoride (PVDF) membranes (Millipore, Germany). The PVDF membranes were sealed with 5% non-fat milk at room temperature for 1 hour, after which we treated them with the appropriate primary antibodies against cyclin D1 (1:5000, Cat#26939-1-Ap, Proteintech), cyclin E1 (1:1000, Cat#11554-1-Ap, Proteintech), BCL-2 (1:5000, Cat#12789-1-Ap, Proteintech), BAX (1:8000, Cat#50599-2-Ig, Proteintech), ADAR1(1:1000, Cat#81284T, CST), CREBZF (1:1000, Cat#ab28700, Abcam), Flag (1:20000, Cat#66008-4-Ig, Proteintech), Myc (1:4000, Cat#16286-1-Ap, Proteintech), His (1:20000, Cat#66005-1-Ig, Proteintech), p-STAT3 (1:1000, Cat#ab267373, Abcam), STAT3 (1:1000, Cat#ab68153, Abcam), and GAPDH (1:50000, Cat#60004-1-Ig, Proteintech) overnight at 4℃. We washed the membranes three times using TBST and then incubated them with HRP-conjugated secondary antibodies for 1 hour at RT. The antigen-antibody reaction was visualized using an enhanced chemiluminescence assay (ECL) kit (Thermo Fisher Scientific, USA).

For Co-IP, cell lysates with overexpressed Flag, Myc, or His tagged fusion proteins were treated with corresponding specific antibodies and magnetic beads for overnight at 4℃. The sediment was washed three times with ice-cold lysis buffer, the precipitates were eluted by boiling in 4×SDS loading buffer. Finally, we resolved the samples on SDS-polyacrylamide gels and performed immunoblotting.

**Cell counting kit-8 (CCK-8) assay and EdU proliferation assay**

To assess the proliferation capacity of breast cancer cells, we performed CCK-8 assay and EdU staining assay following previously described method [4]. For CCK-8 assay, we seeded 5×10^3^ breast cancer cells with the indicated treatments into 96-well culture plates and added 10 μl CCK-8 reagents to each well. The absorbance at 450 nm was measured using a multifunction microplate reader. For the EdU proliferation assay, we added 50 µM EdU to cells after seeding them into 96-well plates and incubated them for 2 hours at 37℃. The cells were then fixed, permeabilized, and stained with EdU. After washing with PBS, the cell nuclei were stained with Hoechst 33342 solution for 15 mins at 37℃ in the dark. We calculated the ratio of EdU-positive stained cells to DAPI-stained cells and acquired images using a fluorescence microscope. We used ImageJ software for statistical analysis.

**Cell apoptosis analysis by flow cytometry and Terminal deoxynucleotidyl transferase-mediated deoxyuridine triphosphate nick-end labeling (TUNEL) assay**

Annexin V Fluorescein Isothiocyanate detection kit (Solarbio, China) was utilized to detect cell apoptosis. In short, breast cancer cells were trypsinized and soft-resuspended in binding buffer. Subsequently, the cells were incubated separately with Annexin V and propidium iodide in the dark for 15 minutes. Finally, the samples were analyzed using a flow cytometer (Beckman, USA).

The In Situ Cell Death Detection Kit (Beyotime, China) was used to perform TUNEL assay on breast cancer cells in the indicated groups as previously described [6]. The TUNEL-positive areas from three fields were quantified using Image J software.

**Biotinylated-RNA pull-down assay and RNA immunopreciptation (RIP) assay**

We performed RNA pull-down assay according to a previously described protocol [7]. In brief, we transcribed circPAPD4 *in vitro* and labeled it with biotin using T7 RNA polymerase (Promega, USA). We incubated the biotinylated circPAPD4 oligo probes with breast cell lysates overnight at 4℃. Afterwards, we added streptavidin magnetic beads to the lysates and incubated them for 1 hour at 37℃. We then washed the beads with wash buffer five times, and isolated the precipitated RNA using the proteinase K-chloroform method for RT-qPCR analysis. The sequences of the oligo probes used are provided in Table S10.

An EZ-Magna RIP RNA bind Protein Immunoprecipitation Kit (Millipore, Germany) was used to conduct RIP assay as described previously [8]. In short, breast cancer cells were digested and then incubated with streptavidin magnetic beads conjugated with either anti-ADAR1 antibodies or negative control IgG. After that, proteinase K was used to extract the immunoprecipitated protein-RNA complex, which was subjected to RT-qPCR analysis.

**Luciferase assay**

To confirm the direct targeting of miR-1269a on circPAPD4 and CREBZF 3'UTR, we designed and synthesized wild type (WT) and mutant (Mut) reporter plasmids for each, which contained the binding sites for miR-1269a. Generay (Shanghai, China) carried out the synthesis. Lipofectamine® 3000 (Invitrogen, USA) was used to co-transfect the reporter plasmids and miR-1269a mimics into BC cells. To perform the STAT3 responsive luciferase reporter assay, we transfected BC cells with CREBZF overexpression or control vector with the 4×M67 pTATA TK-Luc plasmid (STAT3 responsive luciferase reporter). After 48 hours, the Dual Luciferase Reporter Assay kit system (Promega, USA) was used to measure the firefly luciferase and Renilla luciferase activity in the cells.

***In vitro* STAT3 dimerization assay**

The method described previously was modified to measure STAT3 dimerization [9]. In brief, Myc-STAT3 containing cultured cells were lysed at 4°C and mixed with 2×loading buffer (125 mM Tris-HCl, 0.15% bromophenol blue, 20% glycerol, pH 6.8) without β-mercaptoethanol. The proteins were subjected to 10% SDS free polyacrylamide gel electrophoresis, and Myc antibody was used for immunoblotting.

**Preparation of CREBZF mRNA NPs**

To prepare mRNA-encapsulated polymer-lipid hybrid NPs, we used a self-assembly method that was optimized and stabilized based on previously reported study [10, 11]. Firstly, G0-C14 and PLGA were dissolved in dimethylformamide (DMF) at concentrations of 2.5 mg/ml and 5 mg/ml, respectively. Citrate buffer with pH 3.0-3.5 was subsequently added to 250 μg of G0-C14 (in 100 μl of DMF) followed by the addition of 16 μg CREBZF mRNA (in 1 μg/μl of citrate buffer) was added. The solution was gently stirred for 15 min at room temperature to ensure adequate electrostatic complexation. Subsequently, 250 μg PLGA polymers (in 50 μl DMF) was added to the G0-C14/CREBZF mRNA mixture and gently mixed. The resulting nanocomplexes were then added dropwise to 10 ml lipid-PEG aqueous solution (in 1 mg/ml of DNase/RNase-free HyPure water) and stirred at 1000 rpm stirring for 30 min at RT. After being washed three times with ice-cold Hypure water via ultrafiltration device (Millipore, MWCO 100kDa), the organic solvent and free compounds in NPs were removed. Finally, the NPs were concentrated into 1 ml PBS solution for in vitro and in vivo studies.

**Physicochemical features and stability of mRNA NPs**

The mRNA NP sizes were assessed at 20℃ using NanoSIGHT and measured by Nanoparticle Tracking Analysis (NTA). Dynamic light scattering (Brookhaven, USA) and Transmission Electron Microscopy (TEM) were utilized to evaluate the surface charge and morphology of mRNA NPs, respectively. To assess the in vitro stability of mRNA NPs, they were incubated with 10% serum in PBS solution at 37℃ with 100 rpm shaking for 0, 24, 48, 72, 96, and 120 hours. At each time point, an aliquot of the NP solution was taken to determine the particle size and changes in size distribution using NanoSIGHT and NTA.

**Pharmacokinetic (PK) study *in vivo***

To conduct the in vivo PK study, female BALB/c nude mice aged 6 weeks were divided into two groups of three mice each. The first group was administered free Cy5-luc mRNA while the second group received Cy5-luc-mRNA NP through tail vein injection. Blood samples were collected at preset time intervals (0, 0.2, 0.4, 0.8, 1, 2, 4, 8, and 16 hours) from the retro-orbital vein using a heparin-coated capillary tube. The wound was gently pressed for a few seconds to stop bleeding. The Cy5 fluorescence intensity in the collected blood samples was measured with a microplate reader at each time point to describe the PK.

**Biodistribution (BioD) of CREBZF-mRNA-NPs in breast cancer model**

To conduct the BioD study, female nude mice bearing CREBZF-null MCF-7 xenografts were injected with either free Cy5-luc mRNA or Cy5-luc mRNA NP via tail vein. After 24 hours, the tumors and organs were collected and examined using the IVIS Lumina III *In Vivo* Imaging System, and the Cy5 fluorescence intensity was analyzed using Image J software.

**Bioinformation analysis**

To explore circRNA expression profiled in BC, two datasets (GSE182471, consisting of 5 BC tissues and adjacent nontumor tissues; GSE165884, consisting of 4 BC tissues and adjacent nontumor tissues) were downloaded from GEO (<https://www.ncbi.nlm.nih.gov/geo/>). R (version 4.2.1) (https://www.r-project.org/) and limma package were used for difference analysis. Fold change < 0.67 or > 1.5, and *p* < 0.05 were determined as significantly statistical difference [12-15]. TCGA datasets (<https://portal.gdc.cancer.gov/>) were used to explore different expressed miRNA in BC using edgeR package (|log_2_ Fold change| > 1, adjusted p < 0.05). As for the seeking of target gene of miRNA, we used Starbase (<https://starbase.sysu.edu.cn/>), microT_CDS(<https://dianalab.e-ce.uth.gr/html/dianauniverse/index.php?r=microT_CDS>) and miRDB (<https://mirdb.org/>) with following selection criteria: clipExpNum ≥ 2, pancancer num ≥ 7 (Starbase), miTG score ≥ 0.74 (microT-CDS), and Target scor >= 65 (miRDB).

**Reference**

1. Zheng F, Chen J, Zhang X et al. The HIF-1alpha antisense long non-coding RNA drives a positive feedback loop of HIF-1alpha mediated transactivation and glycolysis. Nat Commun 2021; 12: 1341.

2. Jiang Y, Zhao J, Li R et al. CircLRFN5 inhibits the progression of glioblastoma via PRRX2/GCH1 mediated ferroptosis. J Exp Clin Cancer Res 2022; 41: 307.

3. Yang M, Hu H, Wu S et al. EIF4A3-regulated circ_0087429 can reverse EMT and inhibit the progression of cervical cancer via miR-5003-3p-dependent upregulation of OGN expression. J Exp Clin Cancer Res 2022; 41: 165.

4. Wu X, Xiao S, Zhang M et al. A novel protein encoded by circular SMO RNA is essential for Hedgehog signaling activation and glioblastoma tumorigenicity. Genome Biol 2021; 22: 33.

5. Hu Z, Han Y, Liu Y et al. CREBZF as a Key Regulator of STAT3 Pathway in the Control of Liver Regeneration in Mice. Hepatology 2020; 71: 1421-1436.

6. Chen Q, Zhang XH, Massague J. Macrophage binding to receptor VCAM-1 transmits survival signals in breast cancer cells that invade the lungs. Cancer Cell 2011; 20: 538-549.

7. Zheng X, Huang M, Xing L et al. The circRNA circSEPT9 mediated by E2F1 and EIF4A3 facilitates the carcinogenesis and development of triple-negative breast cancer. Mol Cancer 2020; 19: 73.

8. Zhang Y, Li X, Zhang J, Liang H. Natural killer T cell cytotoxic activity in cervical cancer is facilitated by the LINC00240/microRNA-124-3p/STAT3/MICA axis. Cancer Lett 2020; 474: 63-73.

9. Sethi G, Chatterjee S, Rajendran P et al. Inhibition of STAT3 dimerization and acetylation by garcinol suppresses the growth of human hepatocellular carcinoma in vitro and in vivo. Mol Cancer 2014; 13: 66.

10. Xiao Y, Chen J, Zhou H et al. Combining p53 mRNA nanotherapy with immune checkpoint blockade reprograms the immune microenvironment for effective cancer therapy. Nat Commun 2022; 13: 758.

11. Islam MA, Xu Y, Tao W et al. Restoration of tumour-growth suppression in vivo via systemic nanoparticle-mediated delivery of PTEN mRNA. Nat Biomed Eng 2018; 2: 850-864.

12. Marvanova M, Lakso M, Wong G. Identification of genes regulated by memantine and MK-801 in adult rat brain by cDNA microarray analysis. Neuropsychopharmacology 2004; 29: 1070-1079.

13. Wu S, Li Y, Chen S et al. Effect of dietary Astragalus Polysaccharide supplements on testicular piRNA expression profiles of breeding cocks. Int J Biol Macromol 2017; 103: 957-964.

14. Pellatt AJ, Mullany LE, Herrick JS et al. The TGFbeta-signaling pathway and colorectal cancer: associations between dysregulated genes and miRNAs. J Transl Med 2018; 16: 191.

15. Cheng Z, Zhang Y, Wu S et al. Peripheral blood circular RNA hsa_circ_0058493 as a potential novel biomarker for silicosis and idiopathic pulmonary fibrosis. Ecotoxicol Environ Saf 2022; 236: 113451.

| **Supplementary Table S1:**  Effective sequences (5’-3’) of lentivirus plasmids-overexpression. | |
| --- | --- |
| Plasmids | 5’-3’ |
| OE-circPAPD4 | GTAGAAGAATACATGTTCACTTCCAGTGAACAAGAGCATGTTCCCAAACTCAATTTTGGGTCGCCCACCCTTCACTCCAAATCATCAACAACATAATAACTTCTTTACCCTGTCACCTACTGTTTATTCACACCAGCAGCTTATAGATGCACAATTCAACTTTCAGAATGCAGACTTGTCTAGAGCTGTGTCATTACAGCAGCTGACATATGGAAATGTCAGTCCAATACAGACCTCAGCTTCCCCATTATTTCGAGGAAGGAAGAGATTAAGCGATGAAAAAAACCTTCCTCTTGACGGTAAACGGCAACGTTTCCATTCACCCCACCAAGAGCCAACTGTAGTTAACCAGATAGTGCCTTTATCAGGTGAACGAAGATACTCAATGCCACCATTGTTTCATACACATTATGTACCAGATATAGTCAGATGTGTTCCACCTTTTCGAGAAATTGCATTTTTAGAACCTAGAGAAATCACACTGCCTGAGGCCAAAGATAAG |
| OE-CREBZF | GACTTCCGGCTACGCCGTTGTCTGGGTGGCGCGGTCGAGTCATCGCAGGGCCTCACCGCTTCGTTCTCCCGTCCCTCCCCGCGCCTTGGCGCGGGGGGTCGACTAGCCAAGTGAGGCGGGAGGCGACTCGGACCTTTCCCTGCATTTCGTTTCGGCCAGTGCCGGGGGCTACCCGCCCTGGGGCCTGGGATCCTTGGGGCCCGTGAGGCCCACTCTTAGCGGCCGGGGCCTACCGCGGCCCGCCGCTGGCCCTCATGAGGCATAGCCTGACCAAGCTGCTGGCAGCCTCGGGCAGCAACTCCCCAACCCGCAGTGAGAGCCCGGAGCCGGCTGCAACTTGTTCGCTGCCCTCTGACCTGACCCGGGCTGCAGCGGGGGAGGAGGAGACGGCGGCGGCCGGATCTCCCGGCCGCAAGCAGCAGTTTGGCGACGAAGGAGAGTTGGAAGCCGGGAGGGGGAGCCGCGGCGGCGTGGCCGTGCGCGCGCCCTCCCCCGAGGAGATGGAGGAGGAGGCGATCGCCAGCCTCCCGGGGGAAGAGACGGAGGATATGGACTTTCTGTCTGGGCTGGAACTGGCGGATCTCCTGGACCCCAGGCAACCGGACTGGCACCTGGACCCCGGGCTTAGCTCGCCGGGGCCTCTCTCCTCGTCTGGCGGAGGCTCGGATAGCGGCGGCCTGTGGAGAGGGGACGATGACGATGAGGCCGCGGCTGCTGAAATGCAGCGCTTCTCTGACCTGCTGCAAAGGCTGTTAAACGGTATCGGAGGCTGCAGCAGCAGCAGTGACAGTGGCAGCGCCGAAAAGAGGCGGAGAAAGTCCCCAGGAGGAGGCGGCGGTGGCGGCAGCGGTAACGACAACAACCAGGCGGCGACAAAGAGTCCCCGGAAGGCGGCGGCGGCCGCTGCCCGCCTTAATCGACTGAAGAAGAAGGAGTACGTGATGGGGCTGGAGAGTCGAGTCCGGGGTCTGGCAGCCGAGAACCAGGAGCTGCGGGCCGAGAATCGGGAGCTGGGCAAACGCGTACAGGCACTGCAGGAGGAGAGTCGCTACCTACGGGCAGTCTTAGCCAACGAGACTGGACTGGCTCGCTTGCTGAGCCGGCTGAGCGGCGTGGGACTGCGGCTGACCACCTCGCTCTTCAGAGACTCGCCCGCCGGTGACCACGACTACGCTCTGCCGGTGGGAAAGCAGAAGCAGGACCTGCTGGAAGAGGACGACTCGGCGGGAGGAGTCTGTCTCCATGTGGACAAGGATAAGGTGTCGGTGGAGTTCTGCTCGGCGTGCGCCCGGAAGGCGTCGTCTTCTCTTAAAATGTAGGGTCAAGTAATCTGCTCTTTATCCGCGTTTACCCCTTTCAACTCCCTTACACCATGTCAAACTTACCTTAGTGGGACATCTTCACCGGACACATTTCAGAGGAGAGAAAAAAAGTAATATTGAATCTTAAAGTGTTTAGCTAAAAGCATGAATGTGACACAGTAACCAACTCCTAATGATAACATGTGACTATTAAATCTCTCTGACAGTTTCTTTTTTAGGTGATTTCCTTCCTGCCAGGCTCCGTTGTAGGGGTTACAGAACAGTCGTTCCCGCCTCACAACCTGGTAAGGATCCATCTCTTCCCGTAACGCTCATGCTCTGCTGCTTTGTCTACTTTAATGGGCAACATCTCAGTTTGTGTGTGTGTGATTTTTTTTTTTTTTTCTGTTTTGGAAGGTGGGAGGGAAATCTAATTTGGGCCCTGTCCACCCTGGAAACAGACTTGTGCTGGTCAAGAATGTATTTAAGATGCCTCTTCTGGTTGAAATAGCTATTAATGTGTCCCCTTATTCAGACTTGCGTGTACCTAGCTCTTCTGTCCCCAGTGTGGACATGGCCTTGGATGACATCGGTTCCAACTGTACACTGAAACCTGCTTATAGAGAGACAGTTTGGAGACAGTGAAACAGGTGAAGTTGAATGGAAGTTCCGAGTTGTACAAGGTGCAAATTGGAATTCCGATTTTAGGGCAACTTTTCAGAGGTTGACAGTTAAGTATTTGGGGCATGCACAAATGTGATAGTTATTTTGCTGGAGGTGACAGGTATCTCTTAAATATCAACAATGCCTTTTAGTTTTCAGTATGAGATATAGAAACAGTCTATAATGGAGTAACATTTTAATGTCTCACCATTAGAGAAGTTGGAAATTAACATACATATGAGACTCCTGCGTTTTAGTTAATTGGAGAATAAGAATGGCTAATGTTTCTTTGAGGACCAGCAGTGATTACCCTATCACTGAGATTGAATAAATTGTTGAACATCTTTATTTTTGTTGTATTAAAATTTTAGGTTAAATTTATATATGTATGATTAGATATTGAAGGTTGTGAAATGTGAATGAAAACGTGTAAAGTGAGGCTTCACAAAGAATCTTATTCTCTGTGTTTCAAAAGTATTTGTCCTATTTTAAAATAATTTTTTAAATTGAGTGGTTCTGGCCACTTAAATGACACGATTGGTACTATTTTCCTAAGGTGGGATTTATTCCCTTGAATCTATTAACCAGTTGTATAAATTGAATTATGGTGTCCTTCAGTGTAAGAGGCATTAAGAAATTCTTTGTGAAACATCACTGCTTGATAAAGTATATACGTATTTAGCATCCTTGTTTTTCTTTGTGCTAAAGTGGATACAGCTGTTGGGGCAGAAGAGACGGGACCAGCTGCTGGCCACATTTCCTGCTTTATTTTAAAAGGTAGTATAAGAAATGAGGAAAAAGAGGTAATATCAGGGCTTCTGCTGTTTTTTATTTTTAACATGTTCATAATTAAAAAGTATTTTCCAGCAGTCCAAAGATGTAAGTTATCTTACACATAAAATGTTTTATTTTGTTATTTGGTTATGAAAATGGAATCCTTGTTCTTGCACAACTGTAAATGTTTTGTTGCTAGATAATACGATTTGAGACCTGAATTGGTCTTTGGTTTCCAGTGCATCACAGCATATTTTGTAAAATCATCTACTACTGCACTTGAGCATGAATGGGTAGTAGCCAAACTCACAAATTGGAGTGATGAACCTGCTTATACCTAAGGGCAGGAGCAAGCCCCTCACAATGCAGCTGCATGGGTTTTTAGTGCCTACTGAATTATATATATATATACATATATATATATATATATAAACCAAAAGTAGTTGGAAAGATTATTTGAAATGACTAATTTGTGCTATCTTTATGAAATATGTTAAATGTAGCTTTTTTGAAACAGAAGCCTTGAATTGAAATTTAACTAATACTTGAACATTTTGTATATATTTCTTTGTATATAATTTTGTGCAGTACCAATGACAAAAATATGGTGTCATAATAAAACCAGGTTTGTTGATCTTTTAGTTATGGGCTCAAAGAATTTATTCATCTCTAACATGATATTGGAAAATAATGGATGAAAATAGGAAAAATGATTGTTAATGCTGACTGTGGGTCTTAAAAGGTTCTGGAAAGCAGTAAGTTCATTTTTCTAAAAACTATAACATTCTGTTGGAGTATTTTCTTCCTTACGTCAATACTTTTCCTGCATTATTTGAAATTGTGGGCTGGGGAGAAACAGTAGTCAAAGCTTTCTGAATTGAGATACTTTGAAATTCCAAGTGTAGATTTTTAGAATGTCATTTTATAAATGGCAGTTTTTGGAATTACTTGATAAGAACTTTTGAAAATGGAAGGATTAGTATGGCCTATTTTTAAAGCTGCTTTGTTAGGTTCCTTATGTTTTATTAACTGTCTTTTCTCAGTTTCCATTTCATTTTTTTTTTTCTAGTTTTGGTGACTTAGTGATTTTGTCATTTTTTACATCAACTTCATGGTCTTGTTTTTACATGGTAATTGCATGTACTTAGGATCTATCTAATAGGGGCTTTAAATAAATTTGGTCATATTTATGTGTAAGCACATTTTACTGTAAATGTTTGGGTTTCTGAATTTAAACAGATCTGTTTATTTCAGTATGTAGTAAACAATATCTTAAAGTGTCCGATTCACTACTTGTTAATTAAAAAAGTTATGATTAATGTGAAACTGTTGTCTTACTATTTTTAGAAAATTGTGTTCTGGATGATTAGCACATGGATAAAGGAGATTTCTGGAATATAAAATGGATTGTTTTTGAAATTTCTAGGTTTGGCTCTATTTACTGTAATGGTTGAAAACAATTTAGTATTTGGGTGACCCTTTTGTTTTTCTTCTAAATGTGCCTCTGGTAAAATACAGAACTAGACTAAAGATGTAGCTTTTTAATATTTGTCTTTTGATGGTGGCAGGAGTTCATACATTAATTGAACTAACACATCATATTTTGACCTACTATTTCTATCATATTGACTTACTGTTTCTGCACTTCTTTGACCAGACTTATCTTAAAAAGTCCACTTTTGTTAAAATGTAGTCACACCTCTGATATAATCCAACAAGAAGTGTTCAAAATATTTTAAATATTTGTGCATTTTATAATCACTAAATTAATCTCTCTCTCTTCTCTTTGAACAGCTTAGCAGTGTCTGCAAAAACGAATCTTTTCCTACAACCTGTTAACTGACTGGACTGTTGGTAACAAAGTAATTGTGAGAACCATGTCGGTCAAAAATTTGGCATCTGCTGAAAAAAATGAATGCCATTTTCAAGTTCCCAAATTACTTCTATACTGATTTCACTTTCCAGAAATGGAGATATGAAAAGATTCTCTGGAATCCTTGAAAGACTTAATAGAGATACATGAGACTAAGTTAATCTTGGAACAAAATTATACTTTTTTTGTCTTTCATGGGAGTGATACTCAGTTATTGCATATCTTTACAAAATTGTAACCTTTGGAGAATTACAGTATTTCATCAAGAAATTAGAGTTTTTACATAATCAAGAGCATATTCTATGGAATTAACATTTAGTGGACTAGAATTACATAAGCAGGCCATCAGTGATCACAAGGCATATGAGTTTGTATTTATTTCCTGGAACATGAGAGCTAATTTGGAGTACAGACAGACACCCTGCTGAGTAGAATATAAAAGTTAATGTAAAGTTCTGAACTATAAAGAGTAGTCCTTTTAAATAGATGCATTGATTTGTATATCTTTGAAAAAGTTGATTATGATTAAATGTGTGGGTAGTTAATTACAATTTAGGGTAACAACCAGGATGTATGGATTCTTGAGGTTTTGGCTTCTTGAGCTTTCTCTTGAGTACTTGATACTGTTTTCTGAATATTTTGAGGTTTCTAATTAATGGTATGCTAGTTTCTACTGTTTTATATAAAAATTTATCTAGTGTTGTATAAGGACTTTAGTATTGTGGATGATGGGAGTGGCTTTTTGTGGCATATGGTTGTTTTAATTGAAATATTTTTTCAGTTTATTACAAGTGTGAAAAAACACTCAAAACTCTGGATTGTTTTATTTCATTTTTGTTGGATTATGTTAATACATTCCTTCACGTGGTTTAGATTGAGTCATTTTATCTCACTTACTTGGAAATGTGCAGCTGATAGAAGAGGGATTGGGTAGGAGATTGGCTTTTACTTCATAATAAATATACAGTTGGTTATTGTATTATTCAAAACTTATTAAATCCATTCAGTTTCTTACCATTTTAAGTGTATTCAGTGCCTGTGAAGCCAGGCAGCAGAGTATTCCCACTGGCTTAAAACTTAGGCGCTGATGCTCCTACAGTACAGCTAGTTATAATTCCAGAACCAAAGGCACCAACATTATTTGGTGACCTTAACTACTACTTTGATAGAACAGACTGGGTAATTCACTGATAATGAGTCATTACCTTTTTAATATGTGATTATCTAGTTCAGCATTTTGAATGTGTAGTTTAAGGTCTGATTTAGTAAAATTTTAAAGCTAAATAATCTGATGACTCTTGAAAGTTTAATTGGTAGTATGACATTTATGTTTTTTTCCCCCAGACTCACAGCCAATACAAAGTTTTGAGACTCCTAAATGTTAATATTTTTGCTGTTTTTCTGAGCTGTTTTTTTATTTACTTACCACCTAAGCTGCGGATCAAAAAGGGAATTCTTCCATTAGAAATTTGTAGTGTTGTGTGGCCAGCACAGTATTTTTCAGATTTGAATTTCAGTACCTCTGTGTAGCCTCAGTACTCTCAACACCAGTCACTCTTTATTGTCTTAAAATATTCCATACTTTTTGTTGACCCAAAATTGTTGAAAATATTTTGCTCAGTTTTCACAATGAACTAATTGAAAAGTGTAGTTACTGTGTTGTAAACATTTGTGTAGTTCTGTTAATACACTGATTTGAATTTTATTTATCTCAACTTCCTTTAGCGTCTGCCTCCAAATTTTGTCTTTGCTGTATACCCACTGAGATCAATTCCCTAGACCCCTCTGGCTCTTAGTGTACTATAATTTGGGCTTTATAAAATATATTGTCTTAATTTAAATAGAATGTGAGCCCTCTTTTTTTTTTTTTAAGCCAAATAACTACACAAAGTAAATTATAAAAGATTTAAATAGTTTGTCTACAGTGACTAGACTGAAATATAAAAATTGATTGAGGGTAGTTTACTTCAGGTGTTAGCTACAAATGACTAAATGTCCTTAAATAATGCAGATATCTGCCCTCTGAACAAAAATTTTGGCAAAGTAAATTTGTAGCAACAGACTTAACATGATTTATTGCTTATGTTGCCCATTTCTGCTAATGGAAAAGATAATTTAAAAATATGTGGCAGAGGACAGATATTAGAAATACTTTTTGGTTACAGAAACCATTGTAAACTCATCCTCATTTATAAATGGTATTATGCTAGGGAGTAATTGGACTAGGAATGTTTAAAGATAAGTAAGGAAACAATGTGTGCTCAATCGTCAAAGAACTTAGTTATATCATTGGGCTTTGAATTATCTAAGTTGATTACATAATTCTATATTCTTAAGGAGTCTTAACTCGGTACTTGGGTTAACGCCAGAAATTACTTTTAATTCATTGTTTTTTTCACTGGGATGAGTAGAACTCTGCTTTTAGTAAAAGTGACAGAATATAGGTAAAATGGATGATTTGGGATCAGAGCTTTTCATCTTGATATTTTAGCCTGTAAAATAAACAGCTATATTGTTTTGCCCTTTAAA |
| LV-STAT3 | GTCGCAGCCGAGGGAACAAGCCCCAACCGGATCCTGGACAGGCACCCCGGCTTGGCGCTGTCTCTCCCCCTCGGCTCGGAGAGGCCCTTCGGCCTGAGGGAGCCTCGCCGCCCGTCCCCGGCACACGCGCAGCCCCGGCCTCTCGGCCTCTGCCGGAGAAACAGGATGGCCCAATGGAATCAGCTACAGCAGCTTGACACACGGTACCTGGAGCAGCTCCATCAGCTCTACAGTGACAGCTTCCCAATGGAGCTGCGGCAGTTTCTGGCCCCTTGGATTGAGAGTCAAGATTGGGCATATGCGGCCAGCAAAGAATCACATGCCACTTTGGTGTTTCATAATCTCCTGGGAGAGATTGACCAGCAGTATAGCCGCTTCCTGCAAGAGTCGAATGTTCTCTATCAGCACAATCTACGAAGAATCAAGCAGTTTCTTCAGAGCAGGTATCTTGAGAAGCCAATGGAGATTGCCCGGATTGTGGCCCGGTGCCTGTGGGAAGAATCACGCCTTCTACAGACTGCAGCCACTGCGGCCCAGCAAGGGGGCCAGGCCAACCACCCCACAGCAGCCGTGGTGACGGAGAAGCAGCAGATGCTGGAGCAGCACCTTCAGGATGTCCGGAAGAGAGTGCAGGATCTAGAACAGAAAATGAAAGTGGTAGAGAATCTCCAGGATGACTTTGATTTCAACTATAAAACCCTCAAGAGTCAAGGAGACATGCAAGATCTGAATGGAAACAACCAGTCAGTGACCAGGCAGAAGATGCAGCAGCTGGAACAGATGCTCACTGCGCTGGACCAGATGCGGAGAAGCATCGTGAGTGAGCTGGCGGGGCTTTTGTCAGCGATGGAGTACGTGCAGAAAACTCTCACGGACGAGGAGCTGGCTGACTGGAAGAGGCGGCAACAGATTGCCTGCATTGGAGGCCCGCCCAACATCTGCCTAGATCGGCTAGAAAACTGGATAACGTCATTAGCAGAATCTCAACTTCAGACCCGTCAACAAATTAAGAAACTGGAGGAGTTGCAGCAAAAAGTTTCCTACAAAGGGGACCCCATTGTACAGCACCGGCCGATGCTGGAGGAGAGAATCGTGGAGCTGTTTAGAAACTTAATGAAAAGTGCCTTTGTGGTGGAGCGGCAGCCCTGCATGCCCATGCATCCTGACCGGCCCCTCGTCATCAAGACCGGCGTCCAGTTCACTACTAAAGTCAGGTTGCTGGTCAAATTCCCTGAGTTGAATTATCAGCTTAAAATTAAAGTGTGCATTGACAAAGACTCTGGGGACGTTGCAGCTCTCAGAGGATCCCGGAAATTTAACATTCTGGGCACAAACACAAAAGTGATGAACATGGAAGAATCCAACAACGGCAGCCTCTCTGCAGAATTCAAACACTTGACCCTGAGGGAGCAGAGATGTGGGAATGGGGGCCGAGCCAATTGTGATGCTTCCCTGATTGTGACTGAGGAGCTGCACCTGATCACCTTTGAGACCGAGGTGTATCACCAAGGCCTCAAGATTGACCTAGAGACCCACTCCTTGCCAGTTGTGGTGATCTCCAACATCTGTCAGATGCCAAATGCCTGGGCGTCCATCCTGTGGTACAACATGCTGACCAACAATCCCAAGAATGTAAACTTTTTTACCAAGCCCCCAATTGGAACCTGGGATCAAGTGGCCGAGGTCCTGAGCTGGCAGTTCTCCTCCACCACCAAGCGAGGACTGAGCATCGAGCAGCTGACTACACTGGCAGAGAAACTCTTGGGACCTGGTGTGAATTATTCAGGGTGTCAGATCACATGGGCTAAATTTTGCAAAGAAAACATGGCTGGCAAGGGCTTCTCCTTCTGGGTCTGGCTGGACAATATCATTGACCTTGTGAAAAAGTACATCCTGGCCCTTTGGAACGAAGGGTACATCATGGGCTTTATCAGTAAGGAGCGGGAGCGGGCCATCTTGAGCACTAAGCCTCCAGGCACCTTCCTGCTAAGATTCAGTGAAAGCAGCAAAGAAGGAGGCGTCACTTTCACTTGGGTGGAGAAGGACATCAGCGGTAAGACCCAGATCCAGTCCGTGGAACCATACACAAAGCAGCAGCTGAACAACATGTCATTTGCTGAAATCATCATGGGCTATAAGATCATGGATGCTACCAATATCCTGGTGTCTCCACTGGTCTATCTCTATCCTGACATTCCCAAGGAGGAGGCATTCGGAAAGTATTGTCGGCCAGAGAGCCAGGAGCATCCTGAAGCTGACCCAGGTAGCGCTGCCCCATACCTGAAGACCAAGTTTATCTGTGTGACACCAACGACCTGCAGCAATACCATTGACCTGCCGATGTCCCCCCGCACTTTAGATTCATTGATGCAGTTTGGAAATAATGGTGAAGGTGCTGAACCCTCAGCAGGAGGGCAGTTTGAGTCCCTCACCTTTGACATGGAGTTGACCTCGGAGTGCGCTACCTCCCCCATGTGAGGAGCTGAGAACGGAAGCTGCAGAAAGATACGACTGAGGCGCCTACCTGCATTCTGCCACCCCTCACACAGCCAAACCCCAGATCATCTGAAACTACTAACTTTGTGGTTCCAGATTTTTTTTAATCTCCTACTTCTGCTATCTTTGAGCAATCTGGGCACTTTTAAAAATAGAGAAATGAGTGAATGTGGGTGATCTGCTTTTATCTAAATGCAAATAAGGATGTGTTCTCTGAGACCCATGATCAGGGGATGTGGCGGGGGGTGGCTAGAGGGAGAAAAAGGAAATGTCTTGTGTTGTTTTGTTCCCCTGCCCTCCTTTCTCAGCAGCTTTTTGTTATTGTTGTTGTTGTTCTTAGACAAGTGCCTCCTGGTGCCTGCGGCATCCTTCTGCCTGTTTCTGTAAGCAAATGCCACAGGCCACCTATAGCTACATACTCCTGGCATTGCACTTTTTAACCTTGCTGACATCCAAATAGAAGATAGGACTATCTAAGCCCTAGGTTTCTTTTTAAATTAAGAAATAATAACAATTAAAGGGCAAAAAACACTGTATCAGCATAGCCTTTCTGTATTTAAGAAACTTAAGCAGCCGGGCATGGTGGCTCACGCCTGTAATCCCAGCACTTTGGGAGGCCGAGGCGGATCATAAGGTCAGGAGATCAAGACCATCCTGGCTAACACGGTGAAACCCCGTCTCTACTAAAAGTACAAAAAATTAGCTGGGTGTGGTGGTGGGCGCCTGTAGTCCCAGCTACTCGGGAGGCTGAGGCAGGAGAATCGCTTGAACCTGAGAGGCGGAGGTTGCAGTGAGCCAAAATTGCACCACTGCACACTGCACTCCATCCTGGGCGACAGTCTGAGACTCTGTCTCAAAAAAAAAAAAAAAAAAAAGAAACTTCAGTTAACAGCCTCCTTGGTGCTTTAAGCATTCAGCTTCCTTCAGGCTGGTAATTTATATAATCCCTGAAACGGGCTTCAGGTCAAACCCTTAAGACATCTGAAGCTGCAACCTGGCCTTTGGTGTTGAAATAGGAAGGTTTAAGGAGAATCTAAGCATTTTAGACTTTTTTTTATAAATAGACTTATTTTCCTTTGTAATGTATTGGCCTTTTAGTGAGTAAGGCTGGGCAGAGGGTGCTTACAACCTTGACTCCCTTTCTCCCTGGACTTGATCTGCTGTTTCAGAGGCTAGGTTGTTTCTGTGGGTGCCTTATCAGGGCTGGGATACTTCTGATTCTGGCTTCCTTCCTGCCCCACCCTCCCGACCCCAGTCCCCCTGATCCTGCTAGAGGCATGTCTCCTTGCGTGTCTAAAGGTCCCTCATCCTGTTTGTTTTAGGAATCCTGGTCTCAGGACCTCATGGAAGAAGAGGGGGAGAGAGTTACAGGTTGGACATGATGCACACTATGGGGCCCCAGCGACGTGTCTGGTTGAGCTCAGGGAATATGGTTCTTAGCCAGTTTCTTGGTGATATCCAGTGGCACTTGTAATGGCGTCTTCATTCAGTTCATGCAGGGCAAAGGCTTACTGATAAACTTGAGTCTGCCCTCGTATGAGGGTGTATACCTGGCCTCCCTCTGAGGCTGGTGACTCCTCCCTGCTGGGGCCCCACAGGTGAGGCAGAACAGCTAGAGGGCCTCCCCGCCTGCCCGCCTTGGCTGGCTAGCTCGCCTCTCCTGTGCGTATGGGAACACCTAGCACGTGCTGGATGGGCTGCCTCTGACTCAGAGGCATGGCCGGATTTGGCAACTCAAAACCACCTTGCCTCAGCTGATCAGAGTTTCTGTGGAATTCTGTTTGTTAAATCAAATTAGCTGGTCTCTGAATTAAGGGGGAGACGACCTTCTCTAAGATGAACAGGGTTCGCCCCAGTCCTCCTGCCTGGAGACAGTTGATGTGTCATGCAGAGCTCTTACTTCTCCAGCAACACTCTTCAGTACATAATAAGCTTAACTGATAAACAGAATATTTAGAAAGGTGAGACTTGGGCTTACCATTGGGTTTAAATCATAGGGACCTAGGGCGAGGGTTCAGGGCTTCTCTGGAGCAGATATTGTCAAGTTCATGGCCTTAGGTAGCATGTATCTGGTCTTAACTCTGATTGTAGCAAAAGTTCTGAGAGGAGCTGAGCCCTGTTGTGGCCCATTAAAGAACAGGGTCCTCAGGCCCTGCCCGCTTCCTGTCCACTGCCCCCTCCCCATCCCCAGCCCAGCCGAGGGAATCCCGTGGGTTGCTTACCTACCTATAAGGTGGTTTATAAGCTGCTGTCCTGGCCACTGCATTCAAATTCCAATGTGTACTTCATAGTGTAAAAATTTATATTATTGTGAGGTTTTTTGTCTTTTTTTTTTTTTTTTTTTTTTGGTATATTGCTGTATCTACTTTAACTTCCAGAAATAAACGTTATATAGGAACCGTC |

| **Supplementary Table S2:** Effective sequences (5’-3’) of plasmids-knockdown. | |
| --- | --- |
| Plasmids | 5’-3’ |
| sh-NC | UUCUCCGAACGUGUCACGUTT |
| sh-ADAR-1 | GUGAGUUAAUGAAAUACAATT |
| sh-ADAR-2 | GCUUCAACACUCUGACUAATT |
| sh-CREBZF-1 | GCAGCGGUAACGACAACAATT |
| sh-CREBZF-2 | GGAGGAGAGUCGCUACCUATT |

| **Supplementary Table S3:** miRNA mimics and inhibitor sequences (5’-3’). | | |
| --- | --- | --- |
| Name | | 5’-3’ |
| miR-1269a inhibitor | Sense | CCAGUAGCACGGCUCAGUCCAG |
|  | Antisense | None |
| miR-1269a mimic | Sense | CUGGACUGAGCCGUGCUACUGG |
|  | Antisense | AGUAGCACGGCUCAGUCCAGUU |

| **Supplementary Table S4**: The top 50 differentially down-regulated circRNAs in GSE165884. | | | | |
| --- | --- | --- | --- | --- |
| CircRNA | FC | AveExpr | t | P.Value |
| hsa_circ_0001525 | 0.146126136 | 7.76823632 | -8.289139225 | 1.62E-05 |
| hsa_circ_0000380 | 0.18388219 | 6.736948781 | -10.98065071 | 1.57E-06 |
| hsa_circ_0000582 | 0.213752614 | 8.011207141 | -7.695333267 | 2.93E-05 |
| hsa_circ_0000691 | 0.228358326 | 8.114762836 | -3.33406997 | 0.008683524 |
| hsa_circ_0001623 | 0.25360904 | 7.321503742 | -9.221989117 | 6.76E-06 |
| hsa_circ_0001804 | 0.265176628 | 6.0472555 | -9.430790447 | 5.62E-06 |
| hsa_circ_0001275 | 0.276134089 | 10.36428989 | -4.088541067 | 0.002695333 |
| hsa_circ_0000867 | 0.280554709 | 7.183127484 | -5.878013438 | 0.000230997 |
| hsa_circ_0001024 | 0.287375953 | 6.78403125 | -2.543295634 | 0.031425325 |
| hsa_circ_0001745 | 0.303234691 | 8.014267266 | -7.954347407 | 2.25E-05 |
| hsa_circ_0000775 | 0.321855103 | 13.52279491 | -4.208594132 | 0.002253122 |
| hsa_circ_0001085 | 0.386720226 | 7.073014281 | -2.879753758 | 0.01809699 |
| hsa_circ_0001504 | 0.392971645 | 12.67052988 | -4.624682029 | 0.001230195 |
| hsa_circ_0000866 | 0.402843934 | 6.932225281 | -8.860409237 | 9.39E-06 |
| hsa_circ_0001549 | 0.412145712 | 8.42333232 | -5.379445925 | 0.00043754 |
| hsa_circ_0001045 | 0.413374217 | 9.250793688 | -5.566826312 | 0.00034278 |
| hsa_circ_0001916 | 0.423567466 | 10.56736053 | -3.937126776 | 0.003388549 |
| hsa_circ_0001031 | 0.434983174 | 9.842673531 | -4.103257071 | 0.002636483 |
| hsa_circ_0000936 | 0.435586541 | 11.12771111 | -3.616301969 | 0.005559326 |
| hsa_circ_0000295 | 0.450015966 | 8.146385828 | -8.1316876 | 1.89E-05 |
| hsa_circ_0000609 | 0.452197074 | 5.904783172 | -5.251096113 | 0.000518633 |
| hsa_circ_0000324 | 0.458659116 | 11.31086089 | -3.927436854 | 0.003438915 |
| hsa_circ_0000254 | 0.459270284 | 10.40271242 | -3.853428895 | 0.003850723 |
| hsa_circ_0000661 | 0.460148233 | 7.009680391 | -5.799886533 | 0.000254745 |
| hsa_circ_0001444 | 0.461235737 | 5.77977668 | -8.182900491 | 1.79E-05 |
| hsa_circ_0000416 | 0.461348776 | 7.066423148 | -5.69903636 | 0.000289396 |
| hsa_circ_0001463 | 0.461752313 | 9.851840266 | -3.181772756 | 0.01108524 |
| hsa_circ_0000594 | 0.465368582 | 7.406883234 | -7.780609241 | 2.69E-05 |
| hsa_circ_0001516 | 0.470800819 | 7.245702305 | -7.236453873 | 4.77E-05 |
| hsa_circ_0001211 | 0.485982857 | 9.441543164 | -5.708328609 | 0.000285999 |
| hsa_circ_0000524 | 0.48698302 | 11.14524541 | -2.703146859 | 0.024166536 |
| hsa_circ_0000755 | 0.492369541 | 6.053310414 | -5.119791903 | 0.000618663 |
| hsa_circ_0001393 | 0.494347408 | 6.202737602 | -6.171836433 | 0.000161031 |
| hsa_circ_0001545 | 0.495086682 | 6.936374445 | -9.013057148 | 8.17E-06 |
| hsa_circ_0000534 | 0.495544439 | 9.412836547 | -3.410170875 | 0.0076929 |
| hsa_circ_0000590 | 0.496002022 | 5.98825075 | -6.768107803 | 8.01E-05 |
| hsa_circ_0000383 | 0.497063798 | 12.341181 | -2.718138156 | 0.023579276 |
| hsa_circ_0000813 | 0.503086808 | 6.27376057 | -7.209325838 | 4.91E-05 |
| hsa_circ_0001588 | 0.505029192 | 7.968033766 | -1.256336628 | 0.240475544 |
| hsa_circ_0000925 | 0.512734376 | 9.863435328 | -4.172675719 | 0.002376716 |
| hsa_circ_0000423 | 0.514202757 | 7.815430938 | -4.184736608 | 0.002334429 |
| hsa_circ_0001772 | 0.516731627 | 11.09997623 | -1.878395261 | 0.092885422 |
| hsa_circ_0001034 | 0.519341411 | 8.694323992 | -6.274445477 | 0.000142342 |
| hsa_circ_0001842 | 0.521549806 | 12.07221175 | -2.268496427 | 0.049348682 |
| hsa_circ_0000429 | 0.522725645 | 14.87644964 | -3.597580244 | 0.005724591 |
| hsa_circ_0001589 | 0.529574638 | 6.106797188 | -6.90156878 | 6.89E-05 |
| hsa_circ_0001851 | 0.529918795 | 7.222705211 | -3.694469124 | 0.004921606 |
| hsa_circ_0000994 | 0.530714237 | 7.668054969 | -4.357966544 | 0.001807968 |
| hsa_circ_0001704 | 0.531423168 | 6.684308547 | -5.936927359 | 0.00021468 |
| hsa_circ_0000919 | 0.537553453 | 9.786578969 | -4.225214892 | 0.002198262 |

| **Supplementary Table S5**: The top 50 differentially down-regulated circRNAs in GSE182471. | | | | |
| --- | --- | --- | --- | --- |
| CircRNA | FC | AveExpr | t | P.Value |
| hsa_circ_0001204 | 0.25554599 | 13.97891693 | -20.16403961 | 5.89E-10 |
| hsa_circ_0001693 | 0.302484357 | 12.76455636 | -12.48361823 | 8.78E-08 |
| hsa_circ_0001485 | 0.327736087 | 12.40396152 | -8.775851206 | 2.92E-06 |
| hsa_circ_0001605 | 0.327815051 | 13.22044512 | -10.9230966 | 3.39E-07 |
| hsa_circ_0001568 | 0.329956092 | 14.60999278 | -11.0856723 | 2.93E-07 |
| hsa_circ_0000324 | 0.332823673 | 10.78321432 | -5.739019629 | 0.000136771 |
| hsa_circ_0000920 | 0.334113319 | 11.26901566 | -4.520170626 | 0.000899057 |
| hsa_circ_0000281 | 0.347943671 | 6.215923865 | -4.969748172 | 0.000438051 |
| hsa_circ_0000036 | 0.368048353 | 11.24379111 | -4.641545707 | 0.000738383 |
| hsa_circ_0000602 | 0.373544229 | 8.15650677 | -5.961147374 | 9.93E-05 |
| hsa_circ_0000979 | 0.376441879 | 11.76005488 | -5.425415277 | 0.00021761 |
| hsa_circ_0000775 | 0.388316921 | 13.11288594 | -2.563999259 | 0.026556745 |
| hsa_circ_0001184 | 0.38994532 | 8.82845369 | -5.667117526 | 0.000151951 |
| hsa_circ_0000788 | 0.391388112 | 12.48762736 | -6.199160349 | 7.09E-05 |
| hsa_circ_0001631 | 0.397746875 | 5.83909549 | -5.257042286 | 0.00028084 |
| hsa_circ_0000661 | 0.422126981 | 6.373111055 | -9.132023346 | 1.99E-06 |
| hsa_circ_0000141 | 0.426107101 | 10.74370366 | -4.321058219 | 0.001247183 |
| hsa_circ_0001579 | 0.431480181 | 6.2575366 | -1.827887104 | 0.095127274 |
| hsa_circ_0000181 | 0.433210264 | 6.862614925 | -3.982487827 | 0.002201538 |
| hsa_circ_0001766 | 0.442706626 | 8.867495935 | -4.674149321 | 0.000700589 |
| hsa_circ_0001845 | 0.444307032 | 6.55862887 | -3.54094525 | 0.004711442 |
| hsa_circ_0001167 | 0.448823232 | 11.36405979 | -3.719645354 | 0.003454392 |
| hsa_circ_0001140 | 0.451845697 | 7.085444185 | -5.409825001 | 0.000222773 |
| hsa_circ_0000261 | 0.458514531 | 5.40811605 | -4.592530161 | 0.000799294 |
| hsa_circ_0001827 | 0.469248003 | 6.065016665 | -7.782072214 | 9.14E-06 |
| hsa_circ_0000021 | 0.479022743 | 6.43998222 | -4.105578515 | 0.001787667 |
| hsa_circ_0001666 | 0.482693258 | 11.86858311 | -4.366782462 | 0.001156341 |
| hsa_circ_0001104 | 0.484395041 | 9.858698 | -3.517314104 | 0.004909901 |
| hsa_circ_0001644 | 0.488063775 | 9.48525291 | -3.960666539 | 0.002284761 |
| hsa_circ_0000662 | 0.493135576 | 7.95148114 | -2.926419626 | 0.013943002 |
| hsa_circ_0000313 | 0.494124043 | 5.50125366 | -7.916125992 | 7.79E-06 |
| hsa_circ_0001810 | 0.50073486 | 8.89574824 | -3.398604167 | 0.006044934 |
| hsa_circ_0001669 | 0.501156361 | 6.45887462 | -7.660110664 | 1.06E-05 |
| hsa_circ_0001730 | 0.502321155 | 9.97655883 | -3.299858774 | 0.007192354 |
| hsa_circ_0000320 | 0.510028744 | 10.92846437 | -2.94827098 | 0.013411516 |
| hsa_circ_0001599 | 0.51286422 | 6.623658125 | -5.495906864 | 0.000195803 |
| hsa_circ_0000999 | 0.513274193 | 8.782168465 | -4.225680359 | 0.001461577 |
| hsa_circ_0000813 | 0.513451759 | 6.40860921 | -4.584503741 | 0.000809762 |
| hsa_circ_0001690 | 0.523546662 | 6.188863245 | -3.334898029 | 0.006761672 |
| hsa_circ_0000371 | 0.52500939 | 8.620211465 | -2.968731624 | 0.012932341 |
| hsa_circ_0001504 | 0.525516687 | 11.63931672 | -2.426439709 | 0.033872184 |
| hsa_circ_0000919 | 0.533871959 | 11.4096387 | -8.23068721 | 5.39E-06 |
| hsa_circ_0000875 | 0.535971933 | 5.12339552 | -4.70606057 | 0.000665566 |
| hsa_circ_0000288 | 0.544592157 | 8.301129175 | -2.70250414 | 0.020766389 |
| hsa_circ_0001530 | 0.552836448 | 6.65198082 | -4.990552606 | 0.000424006 |
| hsa_circ_0000266 | 0.559980933 | 6.670445015 | -3.125994974 | 0.009781393 |
| hsa_circ_0000065 | 0.562272553 | 7.360472855 | -3.94405212 | 0.002350316 |
| hsa_circ_0001707 | 0.563488429 | 5.856743685 | -3.541535603 | 0.004706591 |
| hsa_circ_0001410 | 0.563765856 | 10.07754273 | -2.873543289 | 0.015318295 |
| hsa_circ_0001412 | 0.565918286 | 13.90244491 | -5.932215388 | 0.000103449 |

| **Supplementary Table S6:** The overlapping down-regulated circRNAs in GSE165884 and GSE182471 and conservation analysis. | |
| --- | --- |
| circRNA name | Multiple Conservation Score |
| hsa_circ_0001504  hsa_circ_0000813  hsa_circ_0000661  hsa_circ_0000324  hsa_circ_0000775  hsa_circ_0000919 | 6.625 |
|  | 3.530 |
|  | 0 |
|  | 0 |
|  | 0 |
|  | 0 |

| **Supplementary Table S7:** Correlations between circPAPD4 expression and clinical characteristics in breast cancer patients (n = 143). | | | | |
| --- | --- | --- | --- | --- |
| **Clinicopathological parameters** | **Total (n=143)** | **CircPAPD4 expression** | | ***P* value** |
|  |  | **Low** | **High** |  |
| **Age (years)** |  |  |  |  |
| ≤ 40 | 24 | 13 | 11 | 0.68 |
| > 40 | 119 | 59 | 60 |  |
| **Menopausal status** |  |  |  |  |
| Premenopausal | 80 | 44 | 36 | 0.21 |
| Postmenopausal | 63 | 28 | 35 |  |
| **Tumor size (cm)** |  |  |  |  |
| ≤ 2 | 66 | 25 | 41 | 0.006** |
| > 2 | 77 | 47 | 30 |  |
| **Lymph node metastasis** |  |  |  |  |
| Negative | 82 | 42 | 40 | 0.81 |
| Positive | 61 | 30 | 31 |  |
| **TNM stage** |  |  |  |  |
| I-II | 123 | 57 | 66 | 0.017* |
| III-IV | 20 | 15 | 5 |  |
| **ER status** |  |  |  |  |
| Negative | 73 | 36 | 37 | 0.8 |
| Positive | 70 | 36 | 34 |  |
| **PR status** |  |  |  |  |
| Negative | 91 | 44 | 47 | 0.53 |
| Positive | 52 | 28 | 24 |  |
| **HER2 status** |  |  |  |  |
| Negative | 96 | 47 | 49 | 0.63 |
| Positive | 47 | 25 | 22 |  |
| **Ki-67 status** |  |  |  |  |
| ≤ 15% | 88 | 51 | 37 | 0.021* |
| > 15% | 55 | 21 | 34 |  |

Note: *p <0.05; **p <0.01;

ER = estrogen receptor, PR = progesterone receptor, HER2 = human epidermal growth factor receptor 2.

| **Supplementary Table S8**. Univariate and multivariate recurrence-free survival analysis of prognostic factors for breast cancer patients (n = 143) | | | | | | |
| --- | --- | --- | --- | --- | --- | --- |
| Clinicopathologic Parameters | Recurrence-free survival | | | | | |
|  | Univariate analysis | | | Multivariate analysis | | |
|  | HR | 95%CI | *p* value | HR | 96%CI | *p* value |
| Age | 0.604 | 0.304-1.196 | 0.148 |  |  |  |
| Menopausal status | 0.627 | 0.339-1.159 | 0.137 |  |  |  |
| Tumor size | 1.426 | 0.783-2.596 | 0.246 |  |  |  |
| Lymph node metastasis | 0.809 | 0.428-1.530 | 0.515 |  |  |  |
| Distance metastasis | 3.458 | 1.221-9.794 | **0.02*** | 1.054 | 0.299-3.722 | 0.934 |
| TNM stage | 2.309 | 1.164-4.584 | **0.017*** | 2.344 | 1.013-5.425 | **0.047*** |
| ER status | 0.815 | 0.449-1.476 | 0.499 |  |  |  |
| PR status | 0.939 | 0.503-1.752 | 0.843 |  |  |  |
| HER2 status | 1.352 | 0.736-2.485 | 0.331 |  |  |  |
| Ki-67 | 2.597 | 1.415-4.766 | **0.002**** | 2.896 | 1.542-5.439 | **0.001**** |
| CircPAPD4 expression | 0.475 | 0.254-0.886 | **0.019*** | 0.414 | 0.216-0.795 | **0.008**** |
| CREBZF expression | 0.350 | 0.183-0.671 | **0.002**** | 0.446 | 0.230-0.866 | **0.017*** |
| **Note:** *p <0.05; **p <0.01; | | | | | | |
| ER, estrogen receptor; PR, progesterone receptor; HER2, human epidermal growth factor receptor 2. | | | | | | |

| **Supplementary Table S9:** The primers (5’-3’) used in this study. | | | |
| --- | --- | --- | --- |
| **Primers** |  | | 5’-3’ |
| **CircPAPD4** | | Forward | GTCAGATGTGTTCCACCTTTTCG |
|  |  | Reverse | ATTTGGAGTGAAGGGTGGGC |
| **PAPD4** | | Forward | ACCTACTGTTTATTCACACCAGC |
|  |  | Reverse | GCCGTTTACCGTCAAGAGGAA |
| **ADAR1** | | Forward | CTGAGACCAAAAGAAACGCAGA |
|  |  | Reverse | GCCATTGTAATGAACAGGTGGTT |
| **miR-1269a** | | Forward | CGGGCCTGGACTGAGCCGTG |
|  |  | Reverse | CAGCCACAAAAGAGCACAAT |
| **miR-124-3p** | | Forward | CGGGCTAAGGCACGCGGTGA |
|  |  | Reverse | CAGCCACAAAAGAGCACAAT |
| **miR-138-5p** | | Forward | CGGGCAGCTGGTGTTGTGAAT |
|  |  | Reverse | CAGCCACAAAAGAGCACAAT |
| **miR-1269b** | | Forward | CGGGCCTGGACTGAGCCATG |
|  |  | Reverse | CAGCCACAAAAGAGCACAAT |
| **MSL2** | | Forward | AGCATCCTAGTGAACTGCTACA |
|  |  | Reverse | TGAGGTTGAAGGTAAAGGGGAA |
| **SPTLC2** | | Forward | AACGGGGAAGTACGGAACG |
|  |  | Reverse | CCCCACATACGTGAGCACAG |
| **ARAP2** | | Forward | GTGTTCAGACATCTAGCCCAC |
|  |  | Reverse | GCGTTTAGGAGGAGACAGCTTA |
| **CREBZF** | | Forward | GACCTGCTGCAAAGGCTGTTA |
|  |  | Reverse | CTGGTTGTTGTCGTTACCGCT |
| **GAPDH** | | Forward | GGACCTGACCTGCCGTCTAG |
|  |  | Reverse | GTAGCCCAGGATGCCCTTGA |

| **Supplementary Table S10:** Sequences (5’-3’) of oligo probes for RNA pull-down. | | |
| --- | --- | --- |
| Name | | 5’-3’ |
| Bio-ctrl | TGCCTGAGGCCAAAGATAAGGTAGAAGAATACATGTTCAC | |
| Bio-circPAPD4 | GTGAACATGTATTCTTCTACCTTATCTTTGGCCTCAGGCA | |

**Supplementary figure legend**

**Figure S1: Screening differentially down-regulated circRNAs in BC.** Venn diagram of top 50 low-expressed circRNAs in breast cancer tissues compared with normal breast tissues in GSE165884 and GSE182471.

**Figure S2: The reverse complementary sequences in the flanking introns of circPAPD4.** The sequence of intron 1 (chr5: 78908899-78915434) was aligned to that of intron 4 (chr5: 78919313-78936673) of the PAPD4 gene by using BLAST (https://blast.ncbi.nlm.nih.gov/Blast.cgi). Highly reverse complementary sequences (81% identity over 598 nucleotides) were found and termed as I1RCM (reverse complementary match in intron 1) and I4RCM (reverse complementary match in intron 4), respectively.

**Figure S3: miR-1269a is upregulated in BC tissues.** Expression of miR-1269a in 20 pairs BC tissues and paracancerous tissues were detected by RT-qPCR. ****p* < 0.001.

**Figure S4: CREBZF is regulated by circPAPD4 and correlated with favorable survival. (A)** Expression of CREBZF in 20 pairs BC tissues and paracancerous tissues were measured by RT-qPCR. **(B)** IHC representative images of CREBZF expression in BC tissues. **(C)** Survival curves illustrated the association between CREBZF expression and recurrence rate of BC patients. **(D)** Expression of CREBZF in BC cells was detected by western blot after transfected with sh-NC, sh-CREBZF-1, and sh-CREBZF-2. **(E)** Expression of CREBZF in BC cells was detected by western blot after co-transfected with EV, OE-circPAPD4, OE-circPAPD4+sh-NC, OE-circPAPD4+sh-CREBZF-1. ****p* < 0.001.

**Figure S5: Knocking out CREBZF in MCF-7 cells.** After designed two sgRNA (sgCREBZF-1, sgCREBZF-2) targeting CREBZF, we constructed Lenti-CAS9-sgRNA plasmids and then transduced into MCF-7 cells. Western blot was used to validate the efficiency of CRISPR-Cas9-mediated genome editing in non-target control (Ctrl-1, Ctrl-2), sgCREBZF-1, and sgCREBZF-2.
